# Alternative exon definition events control the choice between nuclear retention and cytoplasmic export of *U11/U12-65K* mRNA

**DOI:** 10.1371/journal.pgen.1006824

**Published:** 2017-05-26

**Authors:** Jens Verbeeren, Bhupendra Verma, Elina H. Niemelä, Karen Yap, Eugene V. Makeyev, Mikko J. Frilander

**Affiliations:** 1 Institute of Biotechnology, FI-00014 University of Helsinki, Helsinki, Finland; 2 Centre for Developmental Neurobiology, King’s College London, London, United Kingdom; University of Münster, GERMANY

## Abstract

Cellular homeostasis of the minor spliceosome is regulated by a negative feed-back loop that targets *U11-48K* and *U11/U12-65K* mRNAs encoding essential components of the U12-type intron-specific U11/U12 di-snRNP. This involves interaction of the U11 snRNP with an evolutionarily conserved splicing enhancer giving rise to unproductive mRNA isoforms. In the case of *U11/U12-65K*, this mechanism controls the length of the 3′ untranslated region (3′UTR). We show that this process is dynamically regulated in developing neurons and some other cell types, and involves a binary switch between translation-competent mRNAs with a short 3′UTR to non-productive isoforms with a long 3′UTR that are retained in the nucleus or/and spliced to the downstream amylase locus. Importantly, the choice between these alternatives is determined by alternative terminal exon definition events regulated by conserved U12- and U2-type 5′ splice sites as well as sequence signals used for pre-mRNA cleavage and polyadenylation. We additionally show that U11 snRNP binding to the *U11/U12-65K* mRNA species with a long 3′UTR is required for their nuclear retention. Together, our studies uncover an intricate molecular circuitry regulating the abundance of a key spliceosomal protein and shed new light on the mechanisms limiting the export of non-productively spliced mRNAs from the nucleus to the cytoplasm.

## Author summary

The cellular homeostasis of many components of the eukaryotic RNA processing machinery is regulated via negative feed-back pathways that result in the formation of both productive and non-productive mRNA species. Typically, the formation of non-productive mRNAs species results from changes in alternative splicing that disrupt the reading frame of the protein coding region and leads to destabilization of the mRNA. Here, we have investigated the homeostasis regulation of the *U11/U12-65K* mRNA that encodes an essential protein component of the minor (U12-dependent) spliceosome intron recognition complex. We show that homeostasis is regulated at the level of nuclear mRNA export and mRNA 3′-end formation, and that it can be further regulated during neuronal differentiation. We describe a multilayered regulatory system utilizing alternative exon definition interactions that use the input from both spliceosomes and the polyadenylation machinery to decide between productive and non-productive mRNA formation. Because the 65K protein is an essential component of the minor spliceosome, this regulatory pathway can potentially affect the expression of ~700 genes containing U12-type introns.

## Introduction

Export of messenger RNAs from the nucleus to the cytoplasm is a critical step of the eukaryotic gene expression program [1–4]. A strict dependence of this process on proper processing of primary transcripts limits precocious release of immature (pre-)mRNAs from the nucleus and prevents their translation into aberrant and potentially toxic protein products. Three main mRNA processing events have been shown to facilitate recruitment of nuclear export factors to nascent pre-mRNAs. First, a 7-methylguanosine cap is added to the 5′ end of nascent transcripts, which in addition to protecting mRNAs from degradation, promotes their translocation from the nucleus to the cytoplasm [5–8]. Next, exon-junction complexes (EJCs) deposited upstream of the exon-exon junctions that appear after the excision of intronic sequences can function as a binding platform for nuclear export factors [9–11]. Finally, pre-mRNA cleavage and polyadenylation (CP) ensure the stability and translational efficacy of mature mRNAs while at the same time stimulating their nuclear export [12–14]. Notably, splicing and CP machineries often co-operate [15–17] to define the terminal exon in a mutually stimulatory fashion [18–21].

Defects in these processing steps often lead to retention of mRNAs in the nucleus followed by their elimination by corresponding quality control mechanisms [22]. Nuclear retention has been shown to involve the assembly of non-productive U2-type spliceosomal E complexes on intron-containing transcripts [23], which either prevents recruitment of mRNA export factors or alternatively, anchors the pre-mRNA to distinct subnuclear structures. Transcripts with defective 3′ ends are often retained at the transcription site [24] in a manner dependent on the exosomal component Rrp6 [25, 26], and uncleaved β-globin transcripts are associated with a stalled RNA polymerase II on the gene template [26]. In addition, upon induction of osmotic stress, some transcripts undergo decreased termination of transcription and remain bound to chromatin [27]. Besides its quality control function, nuclear retention and elimination of intron-containing transcripts can tune the expression levels of many genes in natural developmental contexts [28–31]. Moreover, some retained transcripts can efficiently escape nuclear decay, offering additional regulatory possibilities. For example, nuclear retention and storage of *CTN* transcripts enables their quick and concerted release to the cytoplasm in response to changing cellular conditions [32].

U12-type introns are a rare type of introns characterized by distinct conserved sequences at their 5′ splice sites (5′ss) and branch point sequences (BPS). Their removal is catalyzed by the so-called minor (or U12-dependent) spliceosome. This complex shares the U5 snRNA and a number of protein factors with the major (or U2-dependent) spliceosome but also contains a set of dedicated snRNAs (U11, U12, U4atac and U6atac) and seven minor spliceosome-specific proteins [33–35]. U12-type introns tend to be spliced either more slowly or less efficiently than the standard U2-type introns and are thought to provide a rate-limiting step modulating gene expression output [36–41]. Mutations in specific components of the minor spliceosome lead to activation of aberrant splice sites or nuclear retention of intron-containing transcripts. Presently, four such human diseases have been described. Of these, Microcephalic Osteodysplastic Primordial Dwarfism Type 1/Taybi-Linder syndrome (MOPD1/TALS; [42–44]) and Roifman Syndrome (RFMN; [45]) have been linked with mutations in the gene encoding U4atac snRNA. Isolated Growth Hormone Deficiency type 1 (IGHD1; [46]) is caused by mutations in the *RNPC3* gene coding for the U11/U12-65K protein, and Myelodysplastic Syndrome (MDS; [47]) by somatic mutations in the *ZRSR2* gene coding for Urp protein involved in 3′ splice site (3′ss) recognition [48].

Previously, we have identified a highly conserved splicing enhancer, termed USSE (U11 snRNP-binding splicing enhancer), which forms the core of a feed-back mechanism regulating the levels of the U11-48K and U11/U12-65K proteins [49]. Both proteins are components of the U11/U12 di-snRNP that mediates the initial recognition of U12-type introns [50, 51]. U11-48K participates in the U12-type 5′ss recognition together with the U11 snRNA [52, 53]. The C-terminus of U11/U12-65K interacts with the U12 snRNA while the N-terminus binds to the U11-snRNP specific 59K protein [54]. Therefore, the U11/U12-65K proteins forms a molecular bridge between the U11 snRNP at the 5′ splice site and the U12 snRNP at the BPS thus connecting opposite ends of U12-type introns [52–54]. The USSE element contains a tandem repeat of U12-type 5′ splice sites. These sites are not used for splicing, but rather activate an upstream U2-type 3′ss by recruiting the U11 snRNP using exon definition interaction between the components of the two spliceosomes [49, 55, 56]. In the case of the *U11-48K* gene (also known as *SNRNP48*), USSE predominantly stimulates inclusion of a “poison” cassette exon leading to nonsense mediated decay (NMD) of its mRNA [49, 56] and a classical negative feed-back regulation of its protein levels. On the other hand, USSE-directed alternative splicing of transcripts derived from the *U11/U12-65K* gene (also known as *RNPC3*) gives rise to an mRNA splicing isoform with an extended 3′UTR (the *65K* long-3′UTR isoform). Because the 65K protein itself is not involved in 5′ss recognition but is rather responsive to the levels of U11 snRNP, this circuitry likely operates in a cross-regulatory manner, similar to the relationship between the major spliceosome-associated proteins U1C and U1-70K [57].

In this study, we show that the *65K* splicing isoform switch is regulated in developing mouse neurons leading to preferential formation of *65K* transcripts with the long 3′UTR that are further associated with transcriptional read-through past the canonical poly(A) site. Association of transcriptional read-through with the *65K* long-3′UTR isoform is also observed in human neuronal samples and some cultured cells including HeLa and HEK293. We use cell lines to show that an evolutionarily conserved, but suboptimal major spliceosome 3′ss located in close proximity to the poly(A) site selectively inhibits *65K* long-3′UTR splicing isoform CP. U1 binding to this site results in read-through transcripts that can be extended to the *AMY2B* locus located ~15 kb downstream. Transcriptional read-through can be explained by a model in which USSE-directed exon definition competes with terminal exon definition interactions that promote activation of the canonical *65K* poly(A) site. Furthermore, we provide evidence that transcripts with the long 3′UTR are retained in the nucleus, whereas the isoform with the short 3′UTR is efficiently exported to the cytoplasm. Nuclear retention of long-3′UTR isoform transcripts requires an intact USSE element and is dependent on exon-definition interactions between components of the minor and major spliceosome. Together, these two mechanisms explain how the U11/U12-65K protein levels, and presumably also the levels of U12-type intron recognition complex, are down-regulated by the USSE element during neuronal differentiation.

## Results

### Developmental regulation of the *U11/U12-65K* isoform pattern

**Fig 1:**
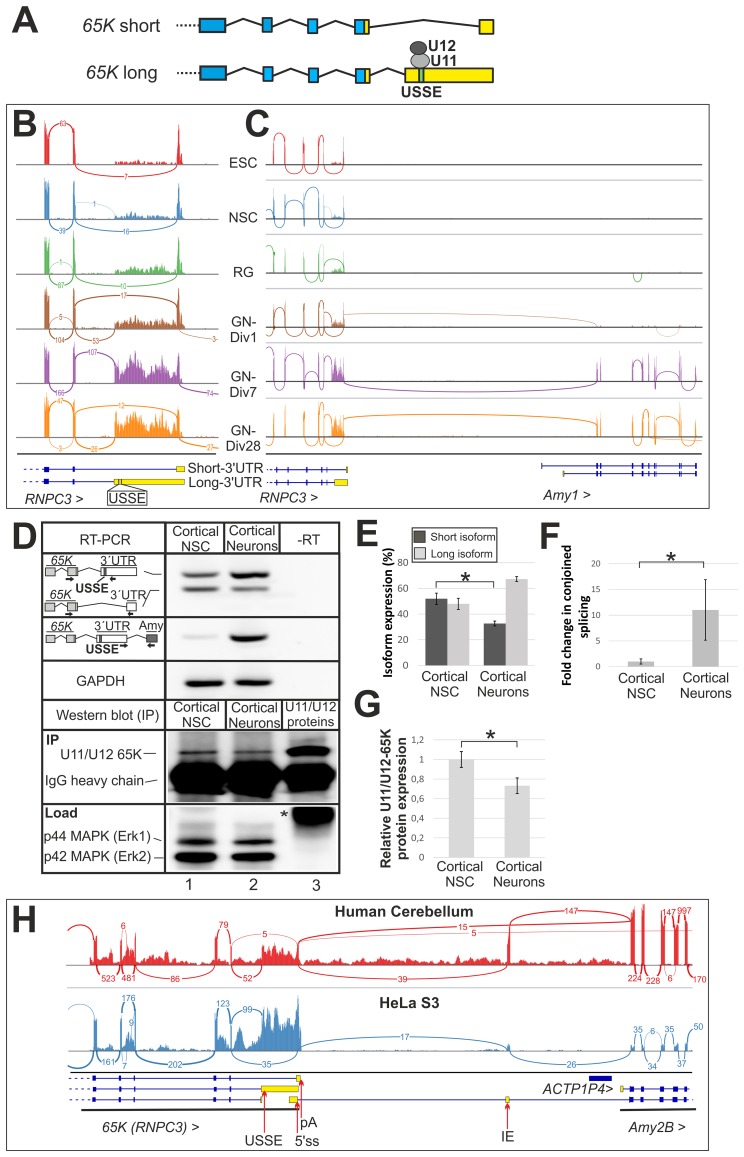
Dynamic regulation of *65K* alternative splicing in developing neuronal cells leads to increase in long-3′UTR isoform expression and transcriptional read-through. **(A)** Model of alternative splicing in the *65K* gene. Binding of U11/U12 di-snRNP to USSE activates alternative splicing to form an isoform with a long 3′UTR. Exons are in blue and the 3′UTR in yellow. **(B)** Sashimi plots of RNAseq data in the 3′ end of the *Rnpc3* locus (*U11/U12-65K*) gene from mouse stem cells (ESC: embryonic stem cells, NSC: neural stem cells), radial glia-type (RG) and glutamatergic neurons (GN) of increasing maturity (DIV1-28). Junction reads are plotted as arcs with the number indicating number of exon-spanning reads. **(C)** Sashimi plot of RNAseq reads in region downstream of the *Rnpc3* (*U11/U12-65K*) gene and in the *Amy1* gene. **(D)** RT-PCR analysis of *Rnpc3* (*65K*) isoform ratio and *Rnpc3-Amy1* conjoined transcripts (upper section) and Western blot analysis (lower section) in mouse cortical neural stem cells and cortical neurons cells. The identities of the amplicons are indicated on left with arrows showing the location of primers. Immunoprecipitation followed by Western blots were performed using a U11/U12 65K antibody (Proteintech, 25820-1-AP) and shown in the panel labeled as "IP". For normalization, p44/42 MAPK (Erk1/2) (Cell Signaling, #4695) were probed in the panel labeled "Load" that represents the sample prior IP. Asterisk indicates the IgG heavy chain. **(E)** RT-PCR quantification of the indicated isoforms from samples shown in (D). Error bars represent the standard deviation of 3 biological replicates and the asterisk indicates p-value < 0.05 in two-tailed Student′s t-test in (E), (F) and (G). **(F)** Fold-change quantification by RT-PCR of conjoined *Rnpc3-Amy1* transcripts from samples shown in (D). Samples were normalized with total *65K* (*Rnpc3)* expression. **(G)** Quantification of the relative U11/U12-65K protein levels from Western blot samples shown in (D). Samples were normalized with p44 MAPK (Erk1) expression. **(H)** Sashimi plot of ENCODE RNAseq reads in the *RNPC3* (*U11/U12-65K*) gene and *AMY2B* gene from human cerebellum and HeLa S3 cells. Junction reads are plotted as arcs with the number indicating number of reads. Arrows indicate position of USSE, 5’ss, pA (poly(A) site) and IE (intergenic exon).

We have previously shown that the splicing enhancer USSE activates an upstream 3′ss in *U11/U12-65K* transcripts through an exon definition-like mechanism [49, 55] resulting in an extended 3′UTR-encoding exon (Fig 1A). This contributes to a negative feed-back expected to generate a relatively stable ratio of the two isoforms in specific cell types and tissues. To ask if this equilibrium could be dynamically regulated during development, we analyzed longitudinal RNAseq data series for mouse embryonic stem cells (ESCs) undergoing differentiation into glutamatergic neurons [58]. The fractional abundance of the long-3′UTR splicing isoform markedly increased during this process (Fig 1B). Concomitantly, we also detected a growing fraction of splice junction reads that connected the U11/U12-65K protein gene *Rnpc3* to the downstream *Amy1* locus encoding a salivary amylase [59] (Fig 1C). Since the difference in 3′ss usage and conjoined *Rnpc3-Amy1* splicing was especially apparent between the neural stem cell and the early neuronal stages, we validated the RNAseq data by RT-PCR analysis using corresponding cell types from developing mouse cortex. This confirmed the short/long splicing isoform dynamics (Fig 1D, upper panel, and Fig 1E) and the increased incidence of conjoined splicing in neurons (Fig 1D and 1F). Notably, we observed a modest (~30%) but significant reduction in the levels of the U11/U12-65K protein in developing neurons as compared to neural stem cells (Fig 1D, lower section, Figs 1G and S1).

The RNA isoform containing these unusual splice junctions appears to emerge as a result of transcriptional read-through of the main *Rnpc3* CP site. Intriguingly, this coincides with the time course of 3′UTR elongation in developing neurons through proximal CP read-through (S2 Fig, [60–63]). The *Rnpc3* transcriptional read-through was not limited to mouse samples since supporting EST and mRNA sequences were available for other mammals (S3 Fig and S1 Table). This pointed at the evolutionary conservation of this event, suggestive of its possible functional importance. Moreover, analysis of the ENCODE RNAseq datasets [64] for human cerebellum and HeLa cells revealed read-through transcripts connecting *RNPC3* with the downstream *AMY2B* locus coding for a pancreatic amylase [65, 66], although HeLa cells showed a lower relative read density in the *Amy2B* locus and intergenic region between the two loci, compared to cerebellum (Fig 1H). The read-through transcripts could also be readily observed in other mammalian cell lines, such as HEK293 and CHO (see below). Consistently, these two loci have also been included in a human conjoined gene database, and the transcriptional read-through has been validated for human brain and pancreas, and described for (unidentified) mouse samples [67]. Interestingly, unlike the situation in mouse where the conjoined transcripts between *Rnpc3* and *Amy1* loci are linked by direct exon-spanning reads where the first available 3′ss within the *Amy1* gene is activated (Fig 1C), in humans, macaque and dog these appear to be predominantly (but not always) linked by divergent species-specific intergenic exons (IEs) situated between the two loci (Figs 1H and S3).

Thus, we have observed developmental regulation of relative *65K* isoform levels in the cells of mouse cortex. At later neuronal differentiation stages, the *65K* long-3′UTR splicing isoform expression is increased concomitantly with the appearance of intergenic transcripts linking the neighboring *Rnpc3* and *Amy1* loci. Such unexpected read-through transcription is also evident in human cells and the cells or tissues of several other mammalian species, suggesting that this evolutionarily conserved process may participate in the regulation of the U11/U12-*65K* (*RNPC3)* gene, thus modulating the activity of the minor spliceosome.

### Transcriptional read-through is linked with long 3′UTR selection while CP promotes accumulation of the short-3′UTR isoform of the *65K* mRNA

**Fig 2:**
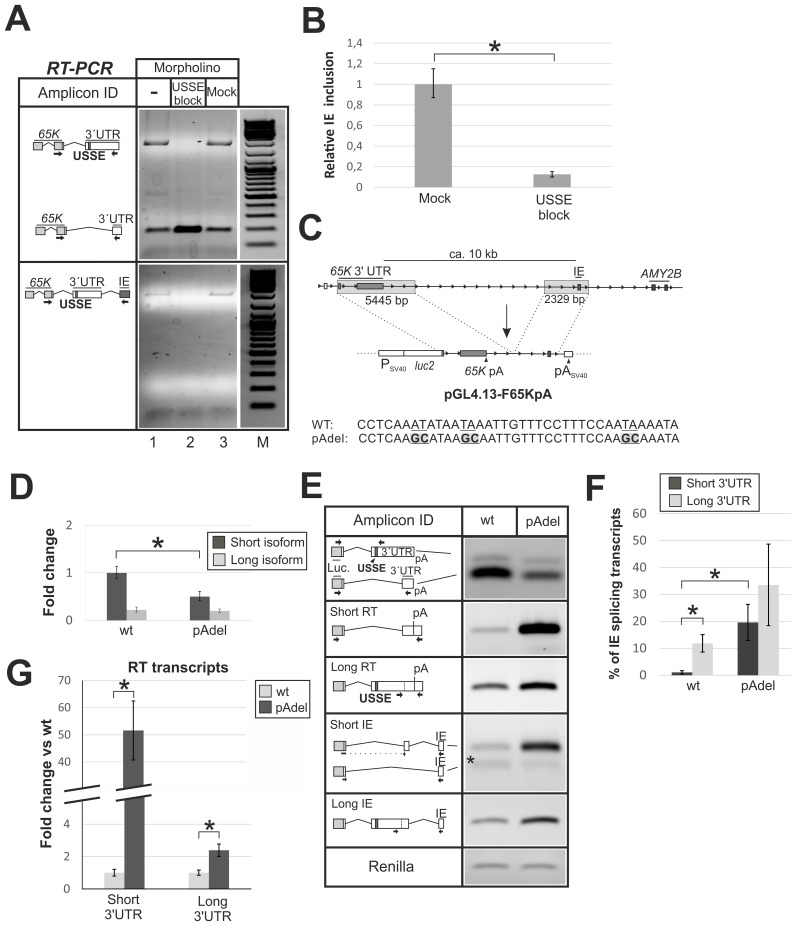
*U11/U12-65K* transcripts with a long 3′UTR are associated with transcriptional read-through. **(A)** Following transfection with mock or block morpholinos in HEK293 cells, RT-PCR was performed to detect both *65K* isoforms simultaneously, using a reverse primer binding at the canonical terminal exon (upper panel), or located in the IE (lower panel). Amplicons were separated on a 1% agarose gel. Endoporter transfection reagent was used for the (-) control sample. The marker (M) is Generuler (Thermo Scientific: #SM0331). **(B)** qRT-PCR analysis of IE expression level for morpholino transfected samples normalized against total *65K* expression. Error bars represent the standard deviation of 3 biological replicates and the asterisk indicates p-value < 0.05 in a two-tailed Student′s t-test in (B), (D), (F) and (G). **(C)** Upper panel. Schematics of the reporter plasmid construction. Shaded boxes depict *65K* sequences cloned into pGL4.13. Lower panel indicate mutations of the putative poly(A) sites (in bold and underlined) of the *65K* gene to generate the pAdel reporter construct. **(D)** CHO cells were transfected with wt and pAdel reporter constructs and construct *65K* long-3′UTR and short-3′UTR splicing isoforms were quantified by qPCR. Fold change is relative to the wt construct *65K* short-3′UTR splicing isoform. **(E)** Multiplex (upper panel) and normal RT-PCR of the samples analyzed in (D) were performed and amplicons separated on 1.3% agarose gel. Primers (shown as arrows) were designed to quantify construct *65K* 3′UTR splicing isoform ratio (upper panel) and read-through and IE splicing of construct *65K* short- and long-3′UTR splicing isoform transcripts. Dashed lines indicate an exon-spanning primer and an asterisk a construct-specific isoform (splicing event could not be found from endogenous *65K* gene) skipping the short-3′UTR specific exon and directly splicing towards to IE. **(F)** qPCR quantification of construct *65K* 3′UTR splicing isoform transcripts that undergo IE splicing from samples analyzed in (E). **(G)** RT-PCR analysis of expression of short- and long-3′UTR splicing isoform transcripts that read-through past the poly(A) site but do not splice to the IE (RT transcripts) from samples analyzed in (E). Fold change is relative to the wt RT transcript expression and samples were normalized against total construct *65K* short- or long-3′UTR splicing isoform levels.

Since the read-through occurred in transformed human cell lines—albeit at a lower level in comparison with differentiated neurons—we used HEK293 cells to investigate the underlying mechanisms. To elucidate a possible mechanistic link between alternative splicing in the *U11/U12-65K* 3′UTR and intergenic splicing/CP read-through, we transfected HEK293 cells with USSE block morpholino oligos that prevent U11/U12 di-snRNP binding to USSE and result in nearly exclusive production of the short-3′UTR splicing isoform [49]. We analyzed the isoform status and the IE inclusion (indicative of the read-though) using RT-PCR with primers capable of amplifying the entire *65K* 3′UTR. In addition to the expected shift to the short-3′UTR splicing isoform in the USSE block samples (Fig 2A, upper panel, lane 2), we also observed a loss of the read-though transcripts in the same sample (Fig 2A, lower panel, lane 2), suggesting that transcriptional read-through is linked to the 3′UTR splicing isoform choice. Consistent with this, qRT-PCR quantification revealed a ~8-fold reduction in IE signal in USSE block reaction compared to the control reaction (Fig 2B). Similarly, by using differential cDNA priming, we found an increase of the long-3′UTR splicing isoform compared to the short-3′UTR splicing isoform when the cDNA is primed from the IE in RT-PCR (S4 Fig). Thus, the USSE-activated long-3′UTR *65K* splicing isoform is more prone to the transcriptional read-through past the canonical poly(A) site and the formation of conjoined *RNPC3-AMY2B* transcripts than its short-3′UTR counterpart.

To test whether the poly(A) site could have a reciprocal effect on the choice between the short and the long 3′UTR-specific 3′ss alternatives, we created a reporter construct containing a luciferase gene fused to genomic DNA spanning from the *65K* 3′UTR to the IE region (Fig 2C). To reduce the size of the construct we deleted a ~6.5 kb fragment between the *65K* 3′UTR and the IE. The highly conserved poly(A) site (see below) and two additional less conserved putative sites were mutated (Fig 2C, pAdel construct), and the constructs were transfected to CHO cells to avoid interference from endogenous human *65K* in our analysis. Analysis by qPCR (Fig 2D) and RT-PCR (Fig 2E, upper panel) revealed a significant decrease in short-3′UTR splicing isoform levels while the long-3′UTR splicing isoform levels were unaffected. Using an established qPCR strategy to quantify the relative incidence of transcript variants [68], we observed that the loss of the poly(A) site led to a ca. 20-fold increase in IE splicing for the short-3′UTR splicing isoform (Fig 2F, 1% vs 20%), while the long-3′UTR splicing isoform showed approximately a 2–3 fold increase (Fig 2F). Additionally, RT-PCR analysis with a reverse primer located downstream of the cleavage site showed a pronounced increase in short-3′UTR splicing isoform transcripts that do not undergo IE inclusion but clearly read through the poly(A) site (read-through, or “RT” transcripts) when the poly(A) is mutated (Fig 2G). On the other hand, the long-3′UTR-specific RT-PCR assay showed only a ~2-fold increase in the RT transcript abundance (Fig 2G).

Taken together, this suggests that the long-3′UTR splicing isoform is associated with transcriptional read-through and formation of conjoined transcripts between the *RNPC3* and *AMY2B* loci and that the presence of a functional poly(A) site promotes the short-3′UTR isoform-specific splicing pattern.

### Utilization of the *65K* CP site is regulated by interaction between U1 snRNP and a conserved splicing donor sequence

**Fig 3:**
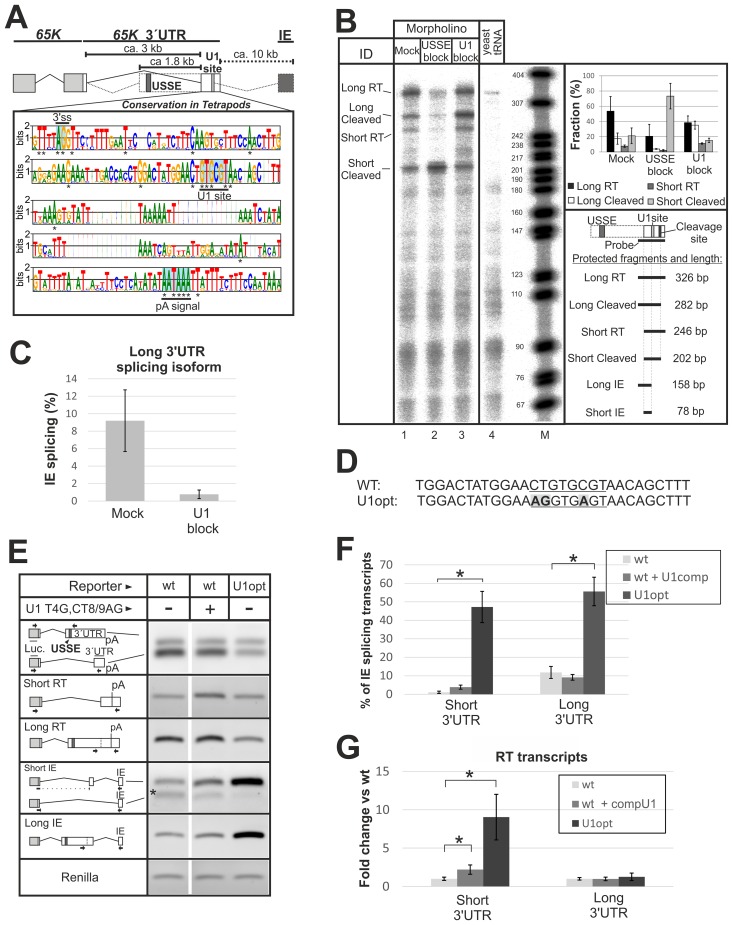
A conserved U1 binding site regulates *65K* cleavage/polyadenylation. **(A)** Sequence logo showing conservation of the region encompassing U1 site and poly(A) signal within the *65K* terminal exon. The data is derived from 30 *Tetrapoda* species. Asterisks indicate 100% sequence conservation for a given position. 3′ss, U1 and poly(A) site are underlined. **(B)** An RNase protection assay was performed on RNA isolated from morpholino treated HEK293 cells 24h after transfection (Mock: targets non-conserved region in *65K* 3′UTR, USSE block: targets USSE, U1 block: targets conserved U1 site). Yeast tRNA served as a control for digested probe and the signal was used for background correction. The marker (M) is pBR322 DNA digested with *Msp*I. Quantification and a key to the different RNase protection fragments are shown on the right. Error bars represent the standard deviation of triplicate samples. **(C)** qPCR quantification of *65K* long-3′UTR splicing isoform transcripts that splice to the IE in HEK293 cells. Error bars represent the standard deviation of triplicate samples. Data could not be obtained for the USSE block sample and the *65K* short-3′UTR isoform (Ct values > 40) **(D)** A *65K* 3′UTR reporter plasmid (see Fig 2C) was mutated (bold and in shade) at the conserved U1 site (underlined) of the *65K* gene to generate a consensus (i.e. “optimal”) U1 site in the U1opt reporter construct. **(E)** CHO cells were transfected with wt (with or without a compensatory mutated U1 snRNA, T4G,CT8/9AG) and U1opt reporter constructs and RT-PCR was performed to analyze constructs *65K* 3′UTR isoform ratio (multiplex) and read-through and IE splicing of *65K* short- and long-3′UTR splicing isoform transcripts. Amplicons were separated on 1.3% agarose gel. Arrows show primer location. Wt samples are from same gel as in Fig 2E. Dashed lines indicate an exon-spanning primer and asterisk a construct-specific isoform (as in Fig 2E and S5 Fig). **(F)** qPCR quantification of construct *65K* 3′UTR splicing isoform transcripts that undergo IE splicing from samples analyzed in (C). Error bars represent the standard deviation of 3 biological replicates and the asterisk indicates p-value < 0.05 in a two-tailed Student′s t-test in (F) and (G). **(G)** RT-PCR analysis of expression of short- and long-3′UTR splicing isoform transcripts that read-through past the poly(A) site but do not splice to the IE (RT transcripts) from samples analyzed in (E). Fold change is relative to the wt RT transcript expression and samples were normalized against total construct *65K* short- or long-3′UTR splicing isoform levels.

A striking feature of the read-through transcripts is the activation of a novel 5′ss that is located ~100 bp upstream of the *65K* poly(A) signal within the shared 3′UTR region present in both isoforms (Fig 3A). The sequence of this element deviates from the consensus 5′ss and is therefore expected to be suboptimal for U1 snRNP binding. Nevertheless, this U1 binding site is conserved in all *Tetrapoda* species (mammals, birds and lizards) examined, pointing at its possible functional significance (Fig 3A). Given that U1 snRNP binding to the last exon is known to suppress cleavage/polyadenylation [69] and, more generally, prevent premature poly(A) site usage [70], we hypothesized that U1 binding to this site may stimulate the transcriptional read-through.

To investigate the effect of the conserved U1 site on the endogenous *65K* CP, we blocked either the USSE element or the U1 binding site in HEK293 cells using corresponding morpholino oligos. A detailed analysis of poly(A) site usage and the splicing isoform ratio using RNase protection confirmed the inhibitory effect of the U1 site on CP in the long-3′UTR splicing isoform (Fig 3B, lane 3) and also the absolute requirement of the USSE for long-3′UTR splicing isoform formation (Fig 3B, lane 2). In the case of the short-3′UTR splicing isoform, the overall level of polyadenylated transcript remained the same as with the mock reaction, but the read-through transcripts showed a slight increase upon U1 site block. Transcripts spliced to the IE were not consistently detected in this assay, suggesting that their levels might be lower compared to polyadenylated and read-through transcripts. Using qPCR analysis, we indeed estimated that ~9% of long-3′UTR splicing isoform transcripts in HEK293 cells undergo splicing towards the IE (Fig 3C). Furthermore, the evolutionarily conserved deviation from the consensus U1 site appears to be necessary for proper functioning of this element because introduction of a consensus U1 site (Fig 3D, U1opt construct) leads to down-regulation of the short-3′UTR splicing isoform (Fig 3E), activation of splicing to the IE (Fig 3E and 3F), and increased read-through past the poly(A) site (Fig 3G) with the short-3′UTR splicing isoform. With the long-3′UTR splicing isoform, we observed additional IE splicing (Fig 3F) but no observable increase in read-through transcription past the poly(A) site (Fig 3G). To confirm this, we co-transfected the WT construct with a plasmid expressing mutated U1 snRNA possessing an optimal base-pairing potential with the conserved 5′ss (U1 T4G,CT8/9AG). Similar to the U1 site mutation, this affected the short-3′UTR splicing isoform only, leading to an increased splicing to the IE and transcriptional read-through. The effect was intermediate compared with the U1opt construct (Figs 3E–3G and S5), most likely due to competition with the endogenous U1 snRNA pool and/or lower expression level of the mutated U1 snRNA.

We conclude that U1 snRNP binding to a conserved but suboptimal 5’ss sequence suppresses CP of *65K* mRNAs containing a long 3′UTR, leading to transcriptional read-through.

### *65K* transcripts with long 3′UTR are retained in the nucleus

The results above indicate that the long-3′UTR *65K* mRNAs but not their short-3′UTR alternatives undergo CP site read-through. Given that 3′-end processing defects often hinder mRNA export from the nucleus to the cytoplasm [12, 24, 26, 71], we hypothesized that the long-3′UTR splicing isoform might be retained in the nucleus. Consistent with this possibility, we showed that the long-3′UTR splicing isoform is less abundant in the cytoplasm than in the nucleus [49], although we were not able to differentiate between accelerated cytoplasmic decay and *bona fide* export problems in this earlier study. Furthermore, the change in the 65K protein levels during neuronal differentiation follows the decline in the short-3′UTR splicing isoform expression rather than the opposite trend observed for the long-3′UTR splicing isoform species (Fig 1D, 1E and 1G; S2 Table) suggesting that the long splicing isoform may be less accessible to the translation machinery.

**Fig 4:**
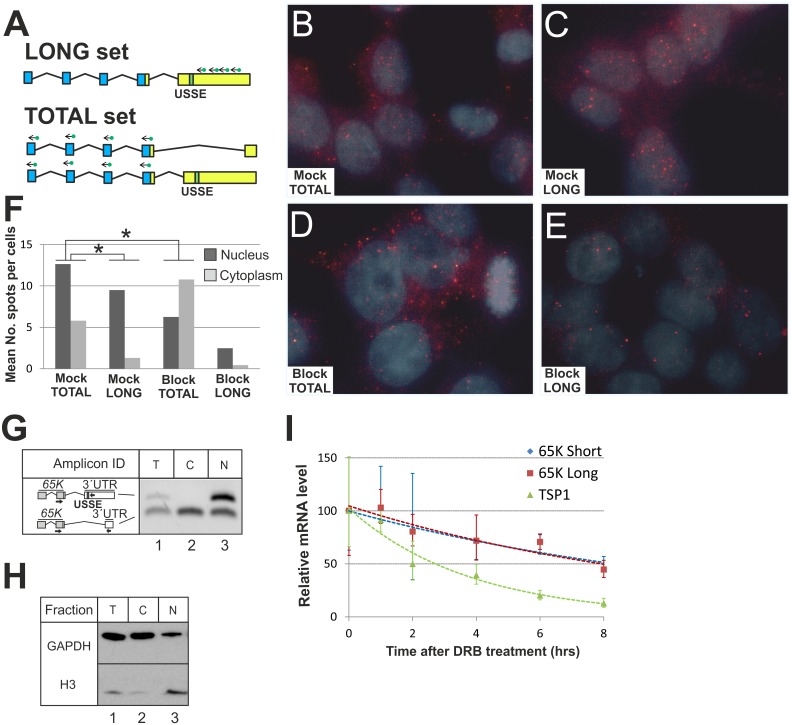
Stable nuclear retention of the *65K* long-3′UTR splicing isoform. **(A)** A schematics showing the location of Cy5-labeled smFISH probes. LONG set: probes bind specifically long-3′UTR isoform, TOTAL set: probes bind both long and short-3′UTR isoform. **(B)** smFISH for mock morpholino treated HEK293 cells with TOTAL probe set and in **(C)** LONG set. **(D)** smFISH for USSE block morpholino treated cells with TOTAL set and in **(E)** LONG set. **(F)** Spots were counted for B (n = 811)), C (n = 346), D (n = 545), and E (n = 140) in the nucleus and cytoplasm using the FISH-QUANT software [97]. Asterisks indicate two-tailed p-value < 0.0001 in Fisher′s test. **(G)** Following cellular fractionation of HEK293 cells, multiplex RT-PCR was performed to measure levels of *65K* long and short-3′UTR isoforms (T: total fraction, C: cytoplasmic fraction, N: nuclear fraction). Black arrows show primer location: forward primer targets both isoforms, while reverse primers target either long or short-3′UTR isoform. **(H)** Western Blot using antibodies against GAPDH and H3 as quality control for cellular fractionation performed in panel G (T: total fraction, C: cytoplasmic fraction, N: nuclear fraction). **(I)**. HEK293 cells were treated with DRB and mRNA levels were assayed at regular time intervals by qRT-PCR for the two *65K* mRNA isoforms and *TSP1* mRNA as indicated. U6 snRNA expression was used for normalization. Error bars represent standard deviation for three biological replicates.

To address this question, we analyzed the subcellular distribution of the two *65K* mRNA splicing isoforms using single molecule fluorescence *in situ* hybridization (smFISH) [72]. Two sets of Cy5-labeled probes were designed: one to detect the long-3′UTR splicing isoform-specific sequences (Fig 4A: LONG) and the other specific to the *65K* protein-coding region and thus shared between the two mRNA splicing isoforms (Fig 4A: TOTAL). Corresponding smFISH analyses of HEK293 cells pre-treated with either a mock morpholino (Fig 4B and 4C) or the USSE-specific morpholino (that biases the splicing pattern towards the short-3′UTR splicing isoform: [49]) (Fig 4D and 4E) revealed that the long-3′UTR splicing isoform was located nearly exclusively in the nucleus (Fig 4C and 4F). Only ~13% of the long-3′UTR splicing isoform-specific spots were detected in the cytoplasm. This was further confirmed by cellular fractionation followed by RT-PCR analyses with isoform-specific primers (Fig 4G and 4H). Nuclear location for the long-3′UTR splicing isoform was also confirmed in HeLa (S6 Fig) and CHO cells (S7 Fig), supporting the generality of this observation. Interestingly, the mRNA spot distribution in the nucleus was uniform, suggesting that the long-3′UTR containing transcripts are able to freely diffuse in the nucleoplasm rather than being retained in a vicinity of their transcription sites (Fig 4C), unlike typical nuclear retention events associated with defects in 3′-end formation [24].

In contrast, smFISH using probes detecting both isoforms revealed a more uniform nuclear-cytoplasmic distribution (Fig 4B). This result is consistent with the earlier observation showing robust cytoplasmic localization for the short-3′UTR splicing isoform. Assuming similar binding and detection efficiencies for both probe sets, we calculated the fraction of short-3′UTR splicing isoform mRNAs in the cytoplasm to be 57%, which agrees with earlier fractionation studies and roughly corresponds to similar data for β-globin [73]. As expected, the incidence of the long-3′UTR spots dramatically decreased in cells pre-treated with the USSE-specific morpholino (Fig 4F: compare panels C and E), accompanied by a concomitant shift in the total mRNA signal from the nucleus to the cytoplasm (Fig 4F).

To ask if the two *65K* mRNA splicing isoforms differed in their stability, we measured their half-lives after treating the cells with DRB (5,6-dichloro-1-β-D-ribofuranosylbenzimidazole), a potent inhibitor of RNA polymerase II elongation [74]. We found that the half-lives of the two isoforms were nearly identical (~10 h: Fig 4I), suggesting that there is no active long-3′UTR splicing isoform-specific decay (neither nuclear nor cytoplasmic). As a control, *TSP1* mRNA showed a robust decay consistent with previous measurements in HEK293 cells (2.3 h; [75]).

We conclude that the long-3′UTR splicing isoform of the *U11/U12-65K* mRNA generated through USSE-stimulated alternative splicing, is relatively long-lived but kept translationally inactive as a result of its nuclear retention. In contrast, the mRNA splicing isoform containing the short 3′UTR is preferentially exported into the cytoplasm where it can program protein synthesis.

### Nuclear retention of the *65K* mRNA requires U11 snRNP binding to its 3′UTR

**Fig 5:**
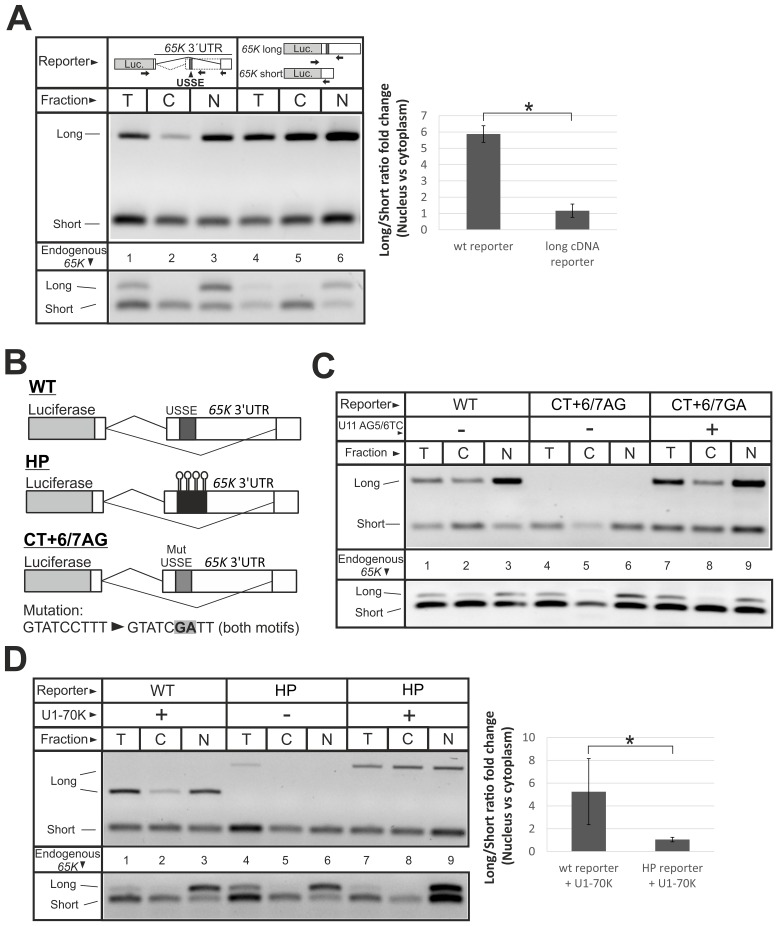
Nuclear retention of *65K* long-3′UTR splicing isoform requires USSE-directed splicing in the 3′UTR. **(A)** HeLa cells were transfected with reporter constructs depicted in the top panels. Following cellular fractionation, the splicing pattern in the 3′UTR was analyzed by multiplex RT-PCR for the constructs and the endogenous *65K*. Arrows indicate primer location. T is total, C is cytoplasmic and N is nuclear fraction. Error bars represent standard deviation for 3 biological replicates and the asterisk indicates p-value < 0.05 in two-tailed Student′s t-test. in (A) and (C). **(B)** Schematics of the reporters used in (C) and (D): 4 BoxB hairpin (HP), wild-type (WT), and the CT+67/GA construct. For the CT+6/7GA construct, mutations (in bold) were made in both U12 5′ss motifs of the USSE. **(C)** HeLa cells were transfected with depicted reporters with, or without co-transfection of U11 snRNA construct carrying compensatory mutations. After cellular fractionation, distribution of construct-derived long and short-3′UTR splicing isoforms was assayed through RT-PCR. Endogenous *65K* isoform cellular distribution served as fractionation quality control. T is total, C is cytoplasmic and N is nuclear fraction. **(D)** HeLa cells were transfected with depicted reporters with, or without co-transfection of λN peptide-fused U1-70K expression construct. After cellular fractionation, distribution of construct-derived long and short-3′UTR splicing isoform was assayed through RT-PCR. Endogenous *65K* isoform cellular distribution served as fractionation quality control. T is total, C is cytoplasmic and N is nuclear fraction.

In search for the underlying mechanism for nuclear retention, we asked whether the long 3′UTR sequence alone contributed to *65K* mRNA retention in the nucleus. Two reporter constructs were made: one where a luciferase is fused to an intron-containing genomic fragment of the *65K* 3′UTR and the other with an intronless cDNA-derived long 3′UTR. The latter reporter was co-transfected with a *65K* short-3′UTR cDNA reporter in equimolar amounts for normalization purposes. Following transfection and cellular fractionation of HeLa cells, RT-PCR showed that the intron-containing long-3′UTR isoform was efficiently retained in the nucleus (Fig 5A). In contrast, the long-3′UTR transcripts expressed from the cDNA-based reporter showed no difference in its subcellular localization compared to the reporter with a cDNA-derived short 3′UTR (Fig 5A). Thus, the sequence of a long 3′UTR alone is not sufficient for the nuclear retention, which also depends on proper post-transcriptional processing (i.e. splicing) of the long-3′UTR isoform.

A major difference between the two *65K* isoforms is that the long 3′UTR contains the USSE element. Given that U1 snRNP binding to mRNA has been reported to lead to nuclear retention [76], we hypothesized that U11 snRNP (or U11/U12 di-snRNP) binding to the USSE element may also promote nuclear retention in addition to its role in regulating alternative splicing. Consistently, a cellular fractionation and RT-PCR analysis revealed nuclear retention of a *U11-48K* mRNA isoform that contains the USSE sequence as a part of a large retained exon (S8 Fig). To ask if base-pairing of U11 snRNA to the USSE is necessary for nuclear retention of the long-3′UTR splicing isoform, we used a genetic rescue strategy described earlier [49]. Briefly, a reporter plasmid with mutated USSE (Fig 5B: CT+67/GA construct) was co-transfected with or without a plasmid expressing U11 snRNA carrying compensatory mutations. We found that expression of mutated U11 snRNA not only restored the expression of the long-3′UTR splicing isoform as expected [49] but this isoform was also preferentially retained in the nucleus (Fig 5C, upper panel; lanes 7–9).

To determine whether nuclear retention was caused by U11 snRNP binding to USSE element or whether the splicing process and the sequence of the long-3′UTR splicing isoform were sufficient for nuclear retention, we created a construct promoting correct splicing of the long-3′UTR isoform in the absence of the USSE element. We replaced the USSE element with four BoxB hairpin sequences (Fig 5B: HP construct) to tether an RS-domain containing U1-70K protein fused to a λN peptide [as described in 55] on the former site of USSE element and scored the upstream 3′ss activation and nuclear retention by cellular fractionation and RT-PCR. Transfection of the HP reporter construct alone showed a low level of 3′ss usage in the absence of λN-70K (Fig 5D, upper panel; lane 4), but 3′ss usage was significantly stimulated upon co-transfection of the λN-70K construct (lane 7). Notably, no preferential nuclear retention was observed for the long-3′UTR splicing isoform mRNA expressed from this construct in the presence of λN-70K (lanes 7–9). In contrast, the transcript with the long 3′UTR was preferentially retained in the nucleus when expressed from the reporter plasmid containing WT USSE (lanes 1–3), or expressed from the endogenous locus (Fig 5D, lower panel). Similarly, co-transfection of the BoxB construct with λN-tagged U11-35K, a functional analog of the U1-70K protein known to promote exon definition [55, 77], also stimulated the upstream 3′ss use but failed to promote nuclear retention of the long-3′UTR splicing isoform (S9 Fig).

Since the BoxB-hairpin array might fold into a bulky structure potentially inhibiting the nuclear retention process, we also replaced the USSE with a more compact purine-rich exonic splicer enhancer (ESE) sequence described previously [78]. Interestingly, in addition to activating the upstream 3'ss, the use of ESE also activated a cryptic 5'ss site downstream of the USSE that was not observed for the wt or hairpin constructs. Importantly, neither of the resulting long-3′UTR splicing isoforms displayed preferential nuclear retention (S10 Fig).

We conclude that nuclear retention of the *65K* long-3′UTR isoform requires binding of the U11 snRNP (or U11/U12 di-snRNP) to its cognate USSE sequence. Since the USSE fails to induce nuclear retention of intronless cDNA-derived transcripts, it is likely that exon definition interactions between USSE and the upstream 3′ss are needed for both activation of the 3′ss and stabilizing the U11 snRNP interaction with *65K* transcripts.

## Discussion

**Fig 6:**
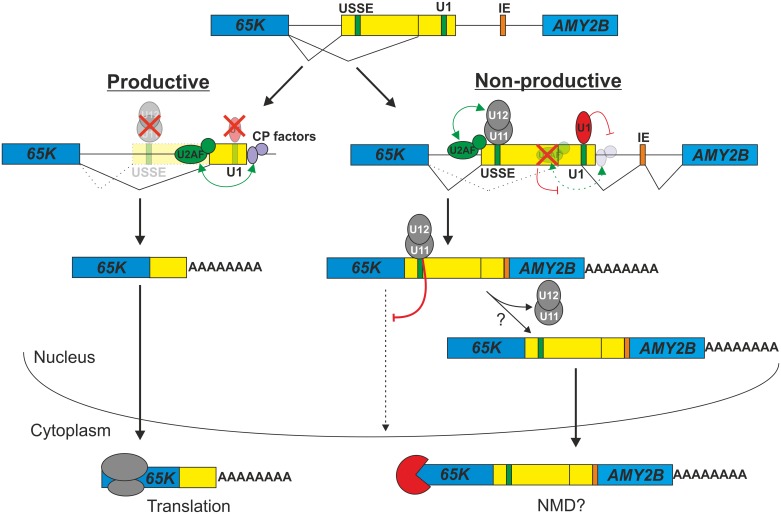
Model for *65K* regulation: Alternative exon definition interactions in the *65K* 3′UTR determine the choice between nuclear export and nuclear retention. Alternative splicing in the *65K* gene generates either a short-3′UTR splicing isoform (productive) or a long-3′UTR splicing isoform (non-productive). For the long-3′UTR isoform, aberrant 3′-end processing together with bound U11 snRNP (or U11/U12 di-snRNP) as a retention factor provide a dual mechanism for nuclear retention. For the short-3′UTR isoform, optimal terminal exon definition interactions overcome U1-mediated inhibition of CP to ensure efficient nuclear export.

In this study, we show that the auto-regulatory feed-back loop that targets the essential U11/U12-65K protein component of the minor spliceosome is itself regulated during neuronal development in a process depending on components of both the minor and the major spliceosomes, as well as the pre-mRNA cleavage/polyadenylation (CP) machinery. We provide evidence that U11 snRNP binding to the ultraconserved USSE sequence has two separate roles in regulating *U11/U12-65K* mRNA levels: it controls alternative splicing to produce an mRNA isoform with a long 3′UTR sequence and promotes retention of this isoform in the nucleus. The formation of the nuclear retention complex depends on successful splicing of an intron upstream of the USSE element suggesting that exon definition interactions are needed to anchor the U11 snRNP on the USSE element. In addition to nuclear retention, the long-3′UTR splicing isoform transcripts are also associated with a bypass of the normal poly(A) site, leading to formation of read-through transcripts that can extend to the downstream *AMY2B* (human) or *Amy1* (mouse) locus. The transcriptional read-through is regulated by a U1 snRNP binding site situated upstream of the normal CP site. Similarly to the USSE element, the U1 site is also evolutionarily highly conserved (Fig 3A), pointing at its functional importance. We propose that these two *cis*-acting signals may act in concert and actively down-regulate the cellular levels of the U11/U12-65K protein during neuronal differentiation by sequestering transcripts with long 3′UTRs into the nuclear compartment and rendering them inaccessible for translation. A model for *U11/U12-65K* post-transcriptional regulation is shown in Fig 6.

Our results indicate that a balance between two distinct exon definition interaction networks, specifically the one between USSE and an upstream 3'ss and another between the poly(A) site and the 3'ss upstream of the terminal exon, determines the structure of the mRNA 3′ end. While binding of the U11 snRNP is an absolute requirement for activation of the long-3′UTR splicing isoform ([49]; Fig 5C]), increased *65K* long-3′UTR splicing isoform expression in developing neuronal cells cannot be explained by corresponding changes in the minor snRNA levels (S11 Fig). Rather, it is likely that changes affecting the CP machinery during neuronal development (reviewed in [79]) contribute to the transcriptional read-through and/or the shift in 3′UTR isoform ratios. Such regulation is thought to lead to transcripts with extended 3′UTRs in mammalian and invertebrate central nervous systems [60–63]. Consistently, the *65K* long-3′UTR splicing isoform activation and the poly(A) site bypass during neuronal differentiation are correlated with distal poly(A) site usage in known examples of alternative CP (S2 Fig). It is also possible that additional factors are involved in the (terminal) exon definition process as suggested for the alternative splicing program regulated by the *48K* USSE [56].

Regardless of the underlying mechanisms, our work shows that a switch towards the long-3′UTR splicing isoform promotes nuclear retention of the *65K* transcripts. Recent studies have described a large number of nuclear mRNA retention events attributed to inefficient pre-mRNA splicing [30, 31, 80]. The USSE-mediated retention events targeting the *48K* and *65K* mRNAs differ from these global events, in that U11 snRNP (or U11/U12 di-snRNP) function as a stand-alone regulator rather than a part of the catalytically active spliceosome [49, 55, 56]. Furthermore, both *48K* and *65K* mRNAs employ an additional mechanism for down-regulation, either NMD-coupled alternative splicing specific for the *48K* mini-exon transcript [56] or transcriptional read-through specific for the *65K* long-3′UTR splicing isoform. Such multilayered inhibitory systems are reminiscent of the *SRSF1* gene where alternative splicing generates six isoforms that are either retained in the nucleus, degraded by NMD or controlled at the translational level [81]. Thus the redundancy in degradation/retention systems with *48K* and *65K* mRNAs might serve as either a fail-safe mechanism or provide extra levels of regulations, for example in a tissue- or developmental stage-specific manner as suggested for *SRSF1* [81]. Consistently, our analysis of mouse neuronal cells indicates that the nuclear retention (*65K* long-3′UTR splicing isoform) and transcriptional read-through may be differentially regulated during differentiation (Fig 1B and 1C). Alternatively or additionally, nuclear retention of isoforms could be regulated so that specific signals (such as cellular stress) release the mRNA into the cytoplasm readily available for translation. This could act on a global fashion as suggested recently [31], or alternatively target specific mRNAs, as has previously been shown for *CTN* RNA that is subjected to post-transcriptional cleavage and polyadenylation to give rise to *mCAT2* mRNA that is released from paraspeckles [32].

### The mechanism of *65K* long-3′UTR isoform nuclear retention

The role of U11/U12 di-snRNP in nuclear retention is consistent with an earlier observation for the major spliceosome where nuclear retention has been described as a mechanism to prevent the escape of partially processed transcripts to the cytoplasm. Specifically, nuclear retention of intron-containing transcripts has been shown to occur when early spliceosomal complexes fail to progress to subsequent catalytic steps [76]. Further work has demonstrated that pre-spliceosomal E complex factors such as U1-70K and U2AF65 can promote nuclear retention for the major spliceosome [23]. Consistently, as the binding of U11/U12 di-snRNP is the earliest stable spliceosomal complex detected for the minor spliceosome, it is possible that two U11/U12 di-snRNPs [55] anchored to the USSE element can mimic unproductive complexes described for the major spliceosome. As the U1-70K and U2AF65 proteins are missing from the minor spliceosome, it is conceivable that analogous protein factors in the U11/U12 di-snRNP complex could be responsible for the inhibition of nuclear export. However, our experiment with tethered U11-35K, a functional U1-70K analog [55, 77], exclude its function as the sole nuclear retention factor (S9 Fig).

The nuclear retention of USSE element-containing *65K* transcripts may be further assisted by inefficient 3′-end formation. In human cells, transcripts with 3′-end processing defects are often retained at the transcription site [24]. Interestingly, uncleaved β-globin transcripts are associated with stalled RNA polymerases and since they do not provide an entry site for exonucleases, their degradation is a relatively slow process [26]. Correspondingly, our experiments in rapidly dividing cells, such as HEK293, show a high incidence of transcripts that read through the poly(A) site but do not splice to the IE in the case of the long-3′UTR *65K* splicing isoform. It is currently unclear whether this fraction represents mRNA where the IE remains unspliced, or whether the defective 3′-end processing has led to stalling of the polymerase. Of note, neither our 3′RACE analysis (S12 Fig) nor RNAseq data such as presented in Fig 1C and 1H were unable to detect any nearby alternative polyadenylation site. In smFISH experiments, retention at the transcription site is characterized by the presence of bright foci in the nucleus [24]. Although such foci can often be seen in our FISH experiments (Fig 4C), a probe set specific for the long-3′UTR isoform shows many mRNAs scattered throughout the nucleus. Presumably, these detectable (and therefore relatively stable) transcripts are released from the transcription site and polyadenylated at either the *65K* poly(A) site (Fig 3B: ~30% of long-3′UTR isoform transcripts are cleaved) or, in the case of the conjoined transcripts (Fig 3C: ~9%), the AMY2B poly(A) site or then terminated between the two loci, particularly with undifferentiated cells. For these transcripts, binding of the U11/U12 di-snRNP at the USSE inhibits nuclear export.

It is possible that the read-through is simply a by-product of the USSE-regulated alternative splicing program which, by promoting the 3′ss choice upstream of the USSE element, also leads to inhibition of CP. However, the high level of evolutionary conservation of the U1 binding site upstream of the poly(A) site and the apparent independent regulation seen during neuronal differentiation argues that the read-through does have a functional significance. Furthermore, activation of the conserved U1 binding site is an evolutionarily conserved process (S3 Fig) further indicating functional significance of transcriptional read-through. Interestingly, many organisms either lack a detectable intergenic exon or employ different IEs to form conjoined transcripts. Thus, the IE inclusion seems mainly a collateral event resulting from binding of U1 snRNP prompting the search for its suitable downstream 3′ss partner. Overall, we conclude that the inefficient processing can function a as "fail-safe" mechanism which may contribute to the nuclear retention of *65K* transcripts or/and render them translation-incompetent as a results of an aberrant 3′UTR structure.

### The conserved U1 site inhibits *65K* long-3′UTR isoform CP

Blocking the U1 with an anti-sense morpholino oligonucleotide promoted CP of the long-3′UTR isoform, suggesting that this conserved element is central in regulating the opposing activities of CP and transcriptional read-through. This is consistent with earlier observations where binding of the U1 snRNP regulated polyadenylation activity, often through direct interactions between the splicing and the CP machinery [69, 82–86]. In U1 inhibition (U1i), U1 snRNP has been shown to exhibit long-distance (> 1000 nt) inhibition of CP, and this inhibition is thought to be due to a disruption of terminal exon definition, rather than to result from an interaction with the U1-70K protein [87]. In the IgM heavy chain gene, again no direct interaction between U1 snRNP and the CP machinery takes place, instead a competition exists between an intronic poly(A) site and an upstream, suboptimal 5′ss [88]. A model has been suggested where a race to form either a cross-intron A-complex with a downstream 3′ss, or the 3′-terminal A-like complex determines the outcome of the competition, and factors that delay (or hasten) this event for splicing promote (or suppress) CP [89]. Similarly, in the case of the *65K* gene, we hypothesize that for the short-3′UTR splicing isoform optimal terminal exon definition interactions overcome U1 snRNP-mediated suppression of CP, whereas for the long-3′UTR splicing isoform, lack of such interactions shift the balance towards U1 snRNP binding. We note that the sequence of the U1 site is highly conserved in evolution but of moderate strength (Fig 3A). Interestingly, mutations changing this to a consensus 5’ss strongly activate splicing to the IE with the short-3′UTR splicing isoform and lead to down-regulation of the mRNA levels (Fig 3E). This argues that the suboptimal 5’ss has been evolutionarily selected to act with the long-3′UTR splicing isoform only.

In conclusion, our data uncover an elaborate gene regulation mechanism engaging components of three major pre-mRNA processing machineries and offering the possibility to integrate a variety of inputs during cell differentiation or in response to other cues. Combined with nuclear retention and formation of conjoined transcripts, this provides an unprecedented example of multi-level post-transcriptional circuitry controlling the expression level of an individual gene. Moreover, since both the *U11/U12-65K* and *U11-48K* genes contain the USSE sequence, are key component of the U12-type intron recognition complex, and are downregulated either at the protein or mRNA levels, respectively, during neuronal differentiation (S2 Table), it is conceivable that this may impact U12-dependent splicing activity. Further studies should elucidate whether the splicing switch to long-3′UTR splicing isoform and the concomitant increase in transcriptional read-through simply dampen *65K* expression levels or form a more intricate feed-back regulatory loop (with other components of the intron-recognition complex) to keep *65K* levels balanced in cells undergoing differentiation. Finally, a previous study has attributed the formation of conjoined transcripts to genomic somatic mutations of poly(A) sites in a small portion of cells [90]. Although hundreds of conjoined genes have been reported, to our knowledge, our study is the first to offer a more detailed mechanistic insight into how they can come into existence under normal physiological conditions.

## Materials and methods

### Ethics statement

All animal procedures were approved by the UK Home Office. Mice were sacrificed in accordance with the UK regulations using increasing concentrations of carbon dioxide or by cervical dislocation.

### Oligonucleotides and plasmids

Oligonucleotides used in this study are listed in S3 Table. For the construction of pGL4.13-F65KpA, human DNA (Life Technologies) was used as a template for two PCRs with primers h65K-221 and h65K-230, and primers h65K-232 and h65K-224, respectively. An overlap-extension PCR was used to fuse these two amplification products, and the resulting PCR product was then cut with *Nde*I and *Mlu*I (Fermentas). This fragment was cloned into a sequence of the pGL4.13-F65K plasmid [49], generated by PCR with primers h65K-219 and h65K-220. pGL4.13-F65KpA was then used as a template for standard site-directed mutagenesis or deletion mutagenesis reactions to construct plasmids used in transfection experiments (U1opt and pAdel). pGL4.13-F65KpA was also used to generate a PCR product that served as a template for in vitro transcription to generate the probe used in the RNase protection assay. Primers h65K-376 and -377 were used for the construction of the ESE *65K* 3′-UTR construct.

### Cell culture and transfection

CHO and HeLa cells were transfected using Lipofectamine 2000 (Invitrogen). Cells were grown in 12-well cell culture dishes and then typically transfected with 100 ng of the *65K* 3′UTR constructs and 1500 ng of pET-41a plasmid (or renilla luciferase expressing plasmid pGL4.73 (Promega) for expression normalization purposes). Morpholino oligos (10 μM) were transfected to HEK293 cells, grown in 12-well cell culture dishes, using the Endoporter reagent (Gene Tools). For the smFISH experiments, transfection in HEK293 cells with morpholino oligos was performed through electroporation using a BioRad GenePulser II at 250 V and a capacitance of 500 μF. For protein analysis, cells were suspended in lysis buffer (25 mM Tris-HCl pH 7.5, 100 mM KCl, 1 mM EDTA, 0.5 mM DTT, protease inhibitor cocktail), sonicated 3 x 30 sec using a Bioruptor sonicator and cleared by centrifugation (Eppendorf 5424R, 13 000 rpm, 10 min). The *65K* mRNA decay experiments were performed in HEK293 cells using 100 μM DRB (5,6-dichloro-1-β-D-ribofuranosylbenzimidazole; Sigma). Dissociated NSC cultures were prepared from E14 mouse embryonic cortices and cultured as neurospheres in NeuroCult Proliferation Kit medium supplemented with 20 ng/ml recombinant human EGF (STEMCELL Technologies). Primary cortical neurons were isolated from E15.5 mouse embryos and cultured as described [91]. Briefly, cortices were dissociated with 2.5% trypsin (Life Technologies) and plated onto a poly-D-lysine (Sigma) treated 6-well plate in Minimum Essential Media (MEM) with L-glutamine (Life Technologies) supplemented with 0.6% glucose (Sigma) and 10% horse serum (Life Technologies).

### Cellular fractionation

After trypsinization, cells were washed with PBS and resuspended in hypotonic buffer (10 mM HEPES-KOH pH 7.9, 1.5 mM MgCl_2_, 10 mM KCl, 0.5 mM DTT, protease inhibitors) followed by 10 min incubation on ice. Cells were collected by centrifugation, resuspended in hypotonic buffer, and lysed with a tight pestle dounce. The lysate was then centrifuged at 230 *g* for 5 min at 4°C, resulting in separation of the nuclear pellet from the cytoplasmic fraction. Nuclei were washed once with hypotonic buffer containing 1 M sucrose. Total and nuclear fractions were sonicated and insoluble material was cleared by centrifugation. Fractionation quality was monitored by western blot with cytoplasmic (GAPDH) and nuclear (H3) markers.

### RT-PCR

RNA was extracted 24 hours after plasmid or morpholino oligo transfection using Trizol reagent (Invitrogen) and was treated with RQ1 DNase (Promega). cDNA was prepared using Revertaid Premium (Thermo Scientific) with either oligo-dT, random primers or gene-specific primers. Standard PCR and multiplex PCR performed as in [49] were carried out using the Phusion polymerase (Thermo Scientific) and amplicons were separated on an agarose gel. PCR cycles were adjusted to ensure detection during the linear phase of amplification. qRT-PCR was performed using a LightCycler 480 Real-Time PCR System (Roche) in 384-well format. Efficiency values of individual qPCR reactions were obtained by the LinRegPCR program [92] and were averaged to the mean efficiency per amplicon. For the calculations of % IE splicing, we used a qPCR quantification method to quantify the relative incidence of transcript variants [68]. In short, the initial number of target molecules N_0_ for each transcript variant (total amount of splicing isoform and amount of splicing isoform that undergoes IE splicing) are calculated and the relative incidence (i.e. % IE splicing for a given splicing isoform) can be obtained using the formula:

$$IE\;splicing\;\%=\frac{N0\left(IE\right)}{N0\left(total\right)}*100\;\left[\%\right]$$ (1)

### Analysis of RNAseq data

Mouse longitudinal RNAseq analysis of mouse embryonic stem cells undergoing differentiation into glutamatergic neurons [58] were downloaded from www.ncbi.nlm.nih.gov/bioproject (accession number PRJNA185305) and aligned to GRCm38/mm10 mouse genome assembly using HISAT2 [93]. RNAseq datasets of human cerebellum (accession number ENCFF730CJE) and HeLa S3 cells (accession number ENCFF343WEZ) were downloaded from www.encodeproject.org/experiments (experiments ENCSR000AEW and ENCSR000CPR, respectively) and aligned to GRCh38/h38. ENSEMBL release 86 transcript annotations were used to streamline identification of known splice junctions. Developmental changes in isoform-specific mRNA expression levels were estimated using Kallisto [94] and a transcriptome index based on the GENCODE M11 GRCm38.p4 release (https://www.gencodegenes.org/mouse_releases/11.html). Transcripts showing monotonic down- or up-regulation trends were identified using the Kendall rank correlation test [95] as implemented in the “Kendall” R package (https://cran.r-project.org/web/packages/Kendall/index.html).

### RNase protection assay

A probe was made from a PCR product generated by using primers h65K-257 and h65K-246 on pGL4.13-F65KpA, followed by T7 RNA polymerase transcription with [α-^32^P]UTP (Perkin Elmer). The RNase protection assay was carried out as a modified version of the assay described in [96]. In short, 30 μg of RNA isolated 24 hours after transfection was treated with DNase and combined with 40 000 cpm of the gel-purified [α-^32^P]UTP-labeled antisense probe. Following ethanol precipitation, the probe was hybridized to the target RNA for 4 hours at 45°C. An RNase solution containing 800 units/mL RNase T1 (Ambion), 17 μg/mL RNase A (Fermentas) and 17 units/mL RNase I (Ambion) was added, and the reaction was incubated for 1 hour at room temperature. Following proteinase K treatment and RNA extraction, the RNA was separated on a 6% denaturing urea-polyacrylamide gel. The gel was then dried, exposed to a phosphor screen and visualized with a phosphoimager (Fuji FLA-5010).

### 3′RACE analysis

3′RACE was employed to analyze poly(A) site usage of the pAdel and wt constructs. For this, a oligo(dT) adapter (h65k-193) was used to prepare cDNA (Revertaid Premium, Thermo Scientific) and subsequently removed by using a QIAquick PCR purification kit. Nested PCR was then performed with Phusion polymerase (Finnzymes) using sequentially reverse primers h65k-194 and h65K-195 (targeting the adapter sequence) and primers h65k-199 and h65k-200. For the initial PCR, 25 cycles were performed and then 1 μl diluted PCR product was used in a nested PCR reaction for an additional 25 cycles, and amplicons were separated on an agarose gel.

### IP and western blot

Whole cell lysate was prepared using NP40 lysis buffer. 50 μg of whole cell lysate was used for immunoprecipitation. Briefly, anti-RNPC3 antibody (Proteintech 25820-1-AP) was added and incubated with rotation at 4°C for 3 hrs. Dynabeads protein G (Life technology, 10004D) were added and incubated further for 1hr at 4°C. Three washes were performed using low salt buffer (20mM Tris-HCl (pH-8), 137 mM NaCl, 10% Glycerol, 1% NP-40, 2mM EDTA). SDS loading dye were added in Dynabeads and sample was heated and loaded on Novex 4–12% Bis-Tris gel (Invitrogen, NP0322). Proteins were transferred on PVDF membrane (Amersham Hybond, 10600023), followed by western blot with anti-RNPC3 antibody. For loading control 5 μg of whole cell lysate was loaded and probed for p44/42 MAPK (Erk1/2) (Cell signaling, 4695) or GAPDH (Cell signaling, 14C10).

### Single-molecule fluorescence *in situ* hybridization

HEK293 cells were grown on Lab-Tek chambered cover glass (with #1 coverglass on the bottom; Thermo Scientific). Custom-made Cy5-labelled Stellaris probes were ordered (Biosearch Technologies, see S4 Table for sequences), and fixation and hybridization were carried out according to manufacturer′s instructions 24 hours after morpholino oligo transfection. Hybridization took place at 37°C for 4 hours. Imaging was performed with the 3I Marianas (3I Intelligent Imaging Innovations) fluorescence microscope and spot distribution was determined using the FISH-QUANT software [97]. The fraction of nuclear *65K* short-3′UTR splicing isoform mRNA for the average HEK293 cell was calculated using the following formula:

$$Sh_{Nuc}=\frac{Tot_{Nuc}\;–\;\left(Lo_{avs}*\;Lo_{Nuc}/Tot_{avs}\right)}{1\;-\;Lo_{avs}/Tot_{avs}}$$ (2)

where:

*Sh*_*Nuc*_ = calculated fraction of short-3′UTR splicing isoform in nucleus

*Tot*_*Nuc*_ = fraction of spots located in nucleus when using TOTAL probe set

*Lo*_*Nuc*_ = fraction of spots located in nucleus when using LONG probe set

*Lo*_*avs*_ = average spots per cell when using LONG probe set

*Tot*_*avs*_ = average spots per cell when using TOTAL probe set

## Supporting information

S1 Fig Western blot analysis of U11/U12 65K. 8 μg of whole cell lysate was separated on 4–12% tris-glycine gel, transferred on PVDF membrane and then probed with U11/U12 65K antibody (Proteintech 25820-1-AP). GAPDH was used as normalization.
(TIF)

S2 Fig Developing neuronal cells show bias towards extended 3′UTR. Sashimi plot of RNAseq reads in mouse *Sugt1*, *Calm1* and *Elav1* genes. RNAseq data is derived from same samples as in Fig 1B and 1C. Junction reads are plotted as arcs.
(TIF)

S3 Fig Conservation of conjoined RNPC3-Amy2B/1 transcripts in mammalian species. A schematic diagram showing alignment of ESTs, mRNAs or RNAseq reads identified from Genbank or from Rat genome database (RGD) and aligned with either human (A) or mouse (B) genomic loci covering 3′ end of *RNPC3* locus and 5′ end of *AMY2B* (panel A) or *AMY1* locus (panel B). The species-specific sequences (orange) are aligned with the gene models on the top. The sequence accession numbers are provided in S1 Table.
(TIF)

S4 Fig Positioning of cDNA primer downstream of poly(A) site decreases short-3′UTR isoform but not long-3′UTR isoform amplification. Multiplex RT-PCR was performed with cDNA from HEK293 RNA. cDNA was made using either primer located upstream of the poly(A) site (α), or on the IE (β and γ) followed by multiplex RT-PCR. Amplicons were separated on a 1.5% agarose gel.
(TIF)

S5 Fig Reduced skipping of short terminal 3′UTR exon after co-transfection of a compensatory mutated U1. Depicted constructs were transfected in CHO cells and short-3′UTR IE splicing pattern was analyzed by RT-PCR. Forward primer was used to allow efficient amplification of the cryptic splicing variant (lower band) depicted with asterisk in Figs 2E and 3E.
(TIF)

S6 Fig Nuclear enrichment of the *65K* long-3′UTR isoform in HeLa cells. Following cellular fractionation of HEK293 cells, multiplex RT-PCR was performed to measure levels of *65K* long and short isoforms (T: total fraction, C: cytoplasmic fraction, N: nuclear fraction). Black arrows show primer location: forward primer targets both isoforms, while reverse primers target either long or short isoform.
(TIF)

S7 Fig Nuclear enrichment of the *65K* long-3′UTR isoform in CHO cells. Following cellular fractionation of CHO cells, qRT-PCR was performed to measure levels of *65K* long and short isoforms. *65K* long versus short isoform ratios were compared in cytoplasmic and nuclear fractions. Error bars represent standard deviation for 3 biological replicates and the asterisk indicates p-value < 0.05 in two-tailed Student′s t-test.
(TIF)

S8 Fig The *48K* long isoform is retained in the nucleus. Following cellular fractionation in CHO cells, qRT-PCR was performed to measure levels of *48K* long and short isoforms. The 4*8K* long isoform contains E5E, an extended exon that results from USSE-directed intron retention. *48K* long versus short isoform ratios were compared in cytoplasmic and nuclear fractions. Error bars represent standard deviation for 3 biological replicates and the asterisk indicates p-value < 0.05 in two-tailed Student′s t-test.
(TIF)

S9 Fig The *65K* long-3′UTR isoform generated through binding of the U11-35K protein alone does not lead to nuclear retention. HeLa cells were transfected with HP reporters and λN peptide-fused U11-35K. After cellular fractionation, distribution of construct-derived long and short isoform was assayed through RT-PCR. T is total, C is cytoplasmic and N is nuclear fraction. Endogenous *65K* isoform cellular distribution served as fractionation quality control.
(TIF)

S10 Fig Replacement of USSE by exon splicing enhancer (ESE) abolishes nuclear retention of long-3′UTR isoform. **(**A) Schematics of the ESE construct, in which the USSE was replaced with an ESE [78]. (B) Sequence of the cryptic splicing isoform with the ESE sequence in bold. **(C)** Multiplex RT-PCR analysis of HeLa cells transfected with the ESE. The identities of the long, short and the cryptic splicing isoforms were confirmed by sequencing.
(TIF)

S11 Fig Levels of minor snRNAs are not increased in different mouse neural cells. Northern blot analysis of indicated mouse cells. One μg of RNA was separated on an 8% polyacrylamide gel, blotted on a nylon filter and sequentially probed for U11, U12 and U4atac snRNAs and major spliceosomal snRNAs, respectively.
(TIF)

S12 Fig 3′RACE analysis does not reveal alternative 65K poly(A) sites. Wt and pAdel constructs were transfected in CHO cells, and 3′RACE was performed to analyze alternative polyadenylation. The pAdel construct served as a negative control. The asterisk indicates a non-specific amplicon as both nested PCR and the pAdel construct did not reveal a similar amplicon. Arrows indicate primer location and the grey arrow the position of the nested reverse primer. The marker (M) is Generuler (Thermo Scientific: #SM0331).
(TIF)

S1 Table EST, mRNA, or RNAseq sequences supporting RNPC3-Amy2A/1 conjoined gene. (DOCX)

S2 Table Minor spliceosome protein-encoding transcripts monotonically down- or up-regulated during neuronal differentiation. (DOCX)

S3 Table List of oligonucleotides used in this study. (DOCX)

S4 Table List of smFISH probes used in this study. (DOCX)
