# Supplementary figures and images for: Alternative exon definition events control the choice between nuclear retention and cytoplasmic export of U11/U12-65K mRNA
Source: PLoS Genet. 2017 May 26;13(5):e1006824. doi: 10.1371/journal.pgen.1006824 (PMC5473595; doi:10.1371/journal.pgen.1006824)

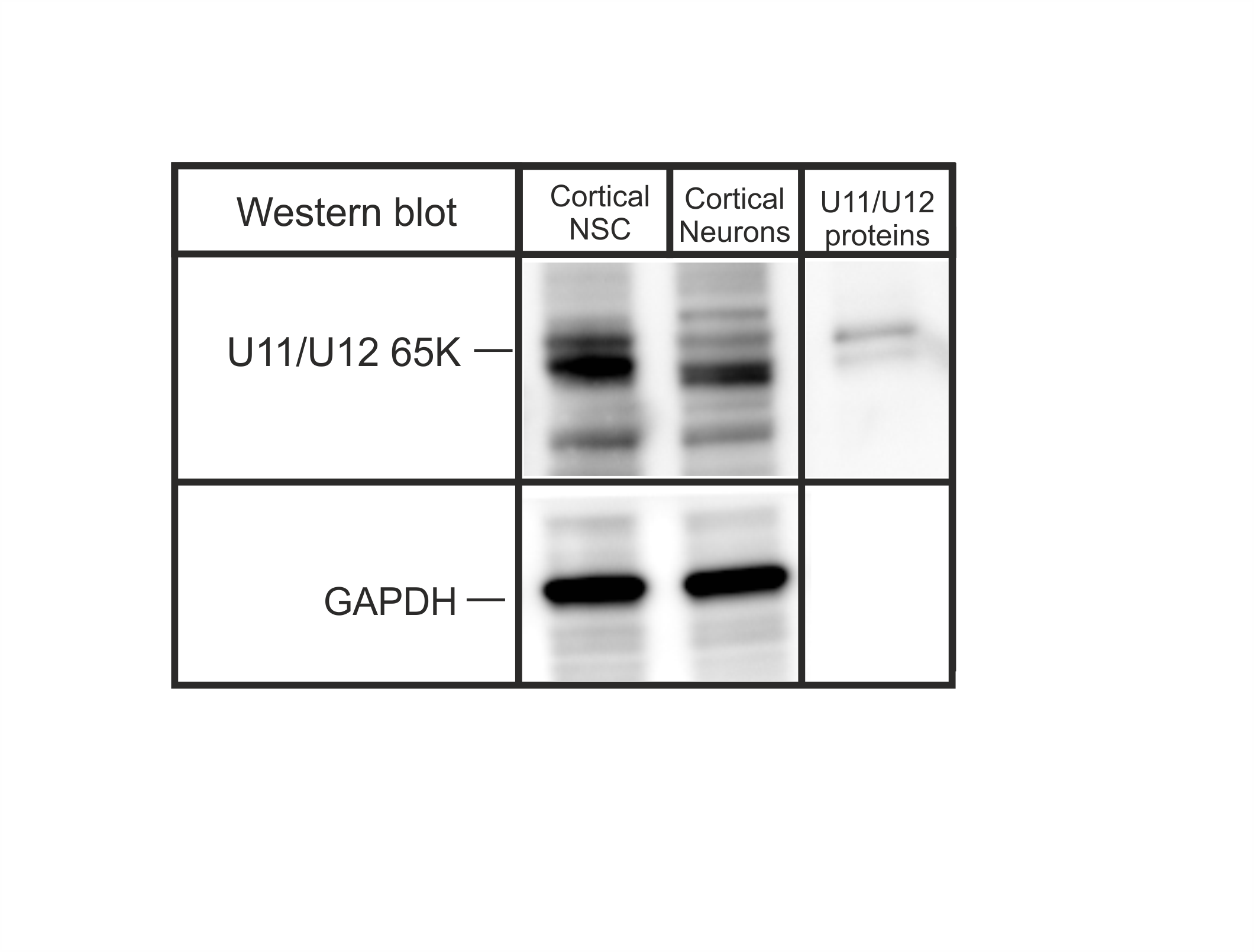

Supplement: S1 Fig — 8 μg of whole cell lysate was separated on 4–12% tris-glycine gel, transferred on PVDF membrane and then probed with U11/U12 65K antibody (Proteintech 25820-1-AP). GAPDH was used as normalization. (TIF) [file pgen.1006824.s001.tif]

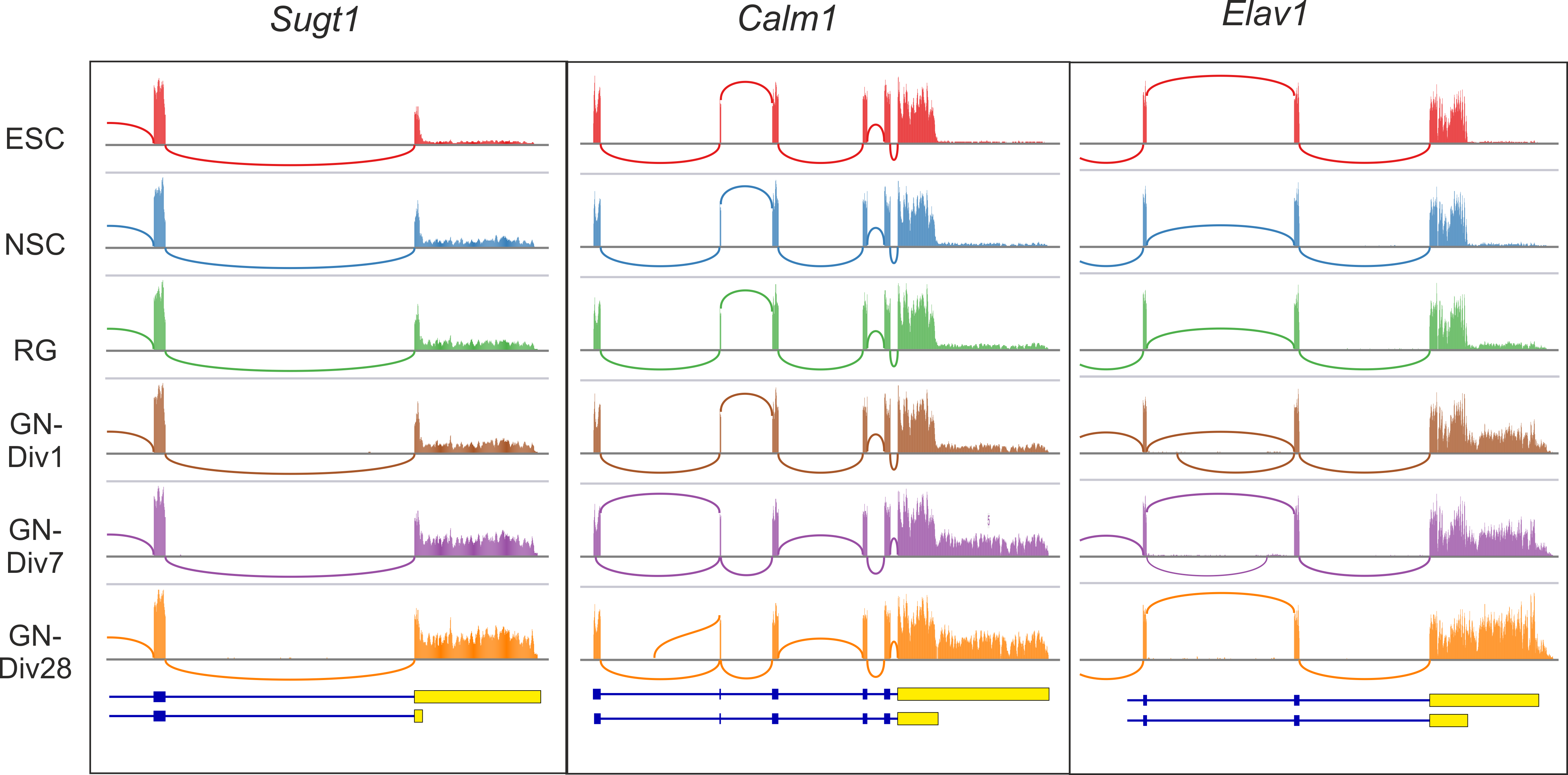

Supplement: S2 Fig — Sashimi plot of RNAseq reads in mouse Sugt1, Calm1 and Elav1 genes. RNAseq data is derived from same samples as in Fig 1B and 1C. Junction reads are plotted as arcs. (TIF) [file pgen.1006824.s002.tif]

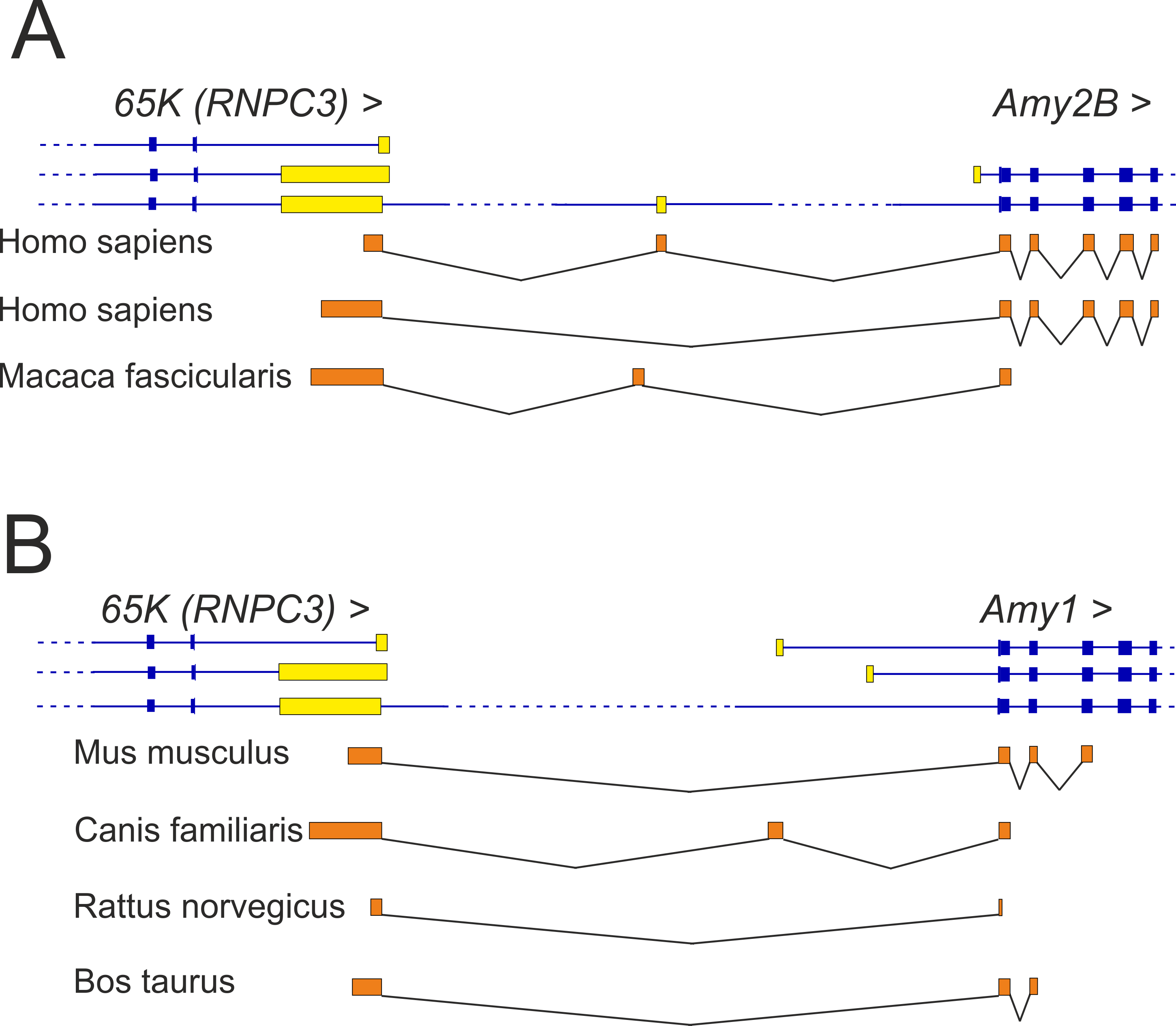

Supplement: S3 Fig — A schematic diagram showing alignment of ESTs, mRNAs or RNAseq reads identified from Genbank or from Rat genome database (RGD) and aligned with either human (A) or mouse (B) genomic loci covering 3′ end of RNPC3 locus and 5′ end of AMY2B (panel A) or AMY1 locus (panel B). The species-specific sequences (orange) are aligned with the gene models on the top. The sequence accession numbers are provided in S1 Table. (TIF) [file pgen.1006824.s003.tif]

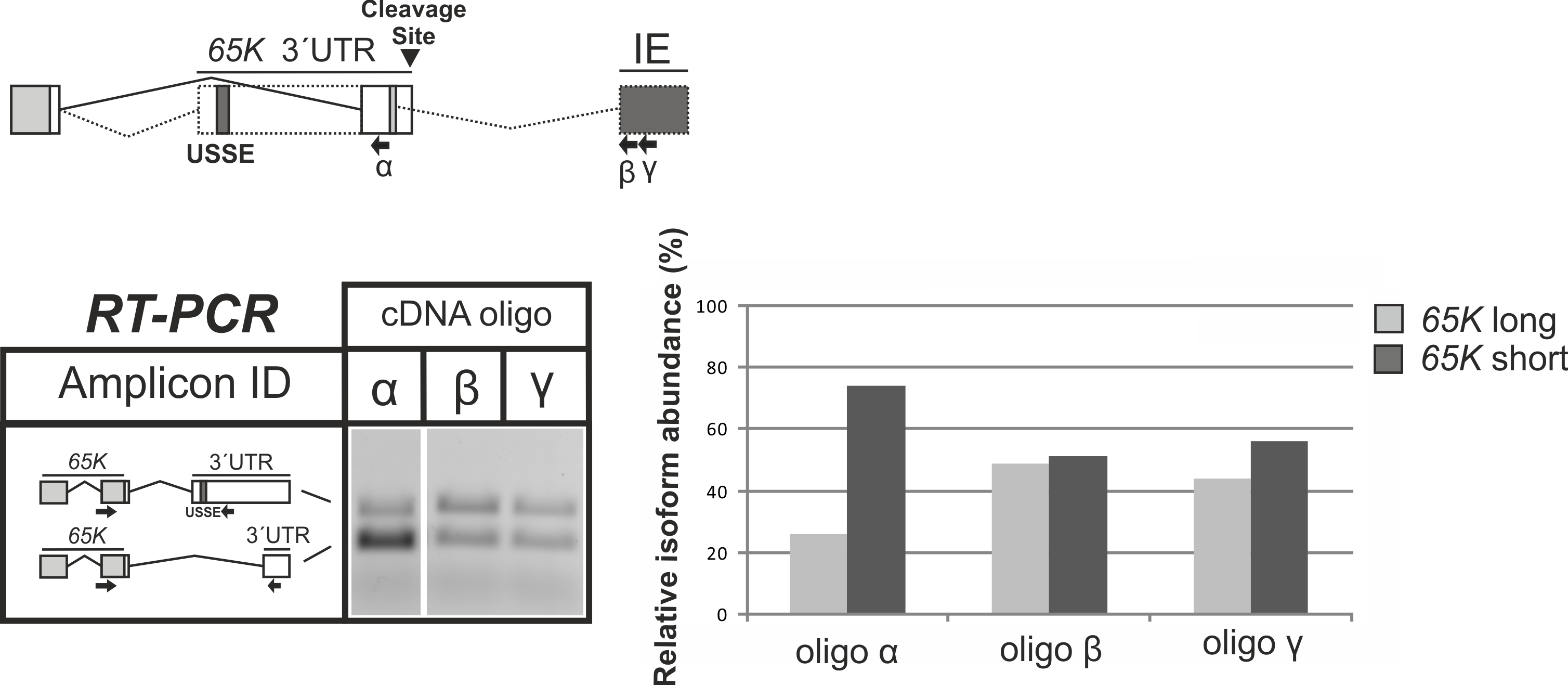

Supplement: S4 Fig — Multiplex RT-PCR was performed with cDNA from HEK293 RNA. cDNA was made using either primer located upstream of the poly(A) site (α), or on the IE (β and γ) followed by multiplex RT-PCR. Amplicons were separated on a 1.5% agarose gel. (TIF) [file pgen.1006824.s004.tif]

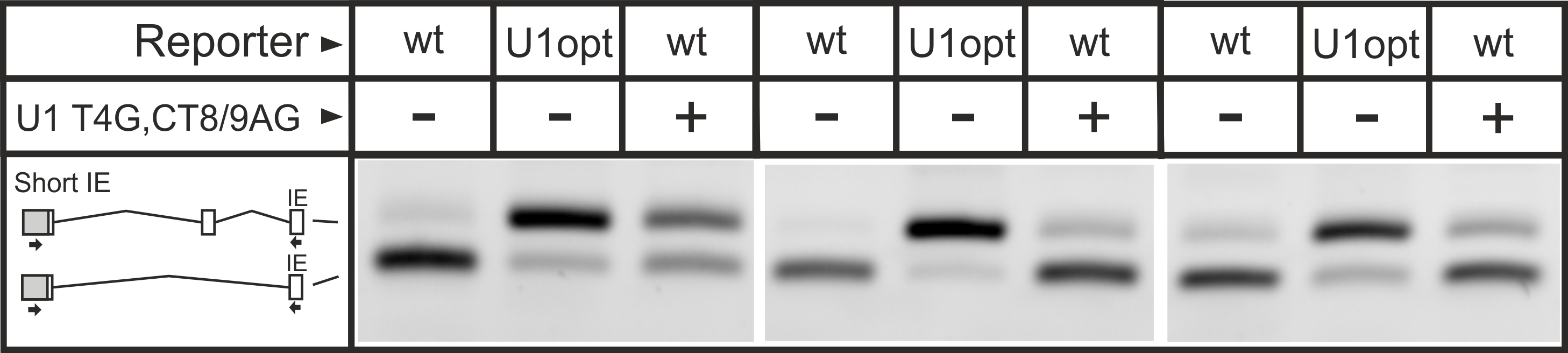

Supplement: S5 Fig — Depicted constructs were transfected in CHO cells and short-3′UTR IE splicing pattern was analyzed by RT-PCR. Forward primer was used to allow efficient amplification of the cryptic splicing variant (lower band) depicted with asterisk in Figs 2E and 3E. (TIF) [file pgen.1006824.s005.tif]

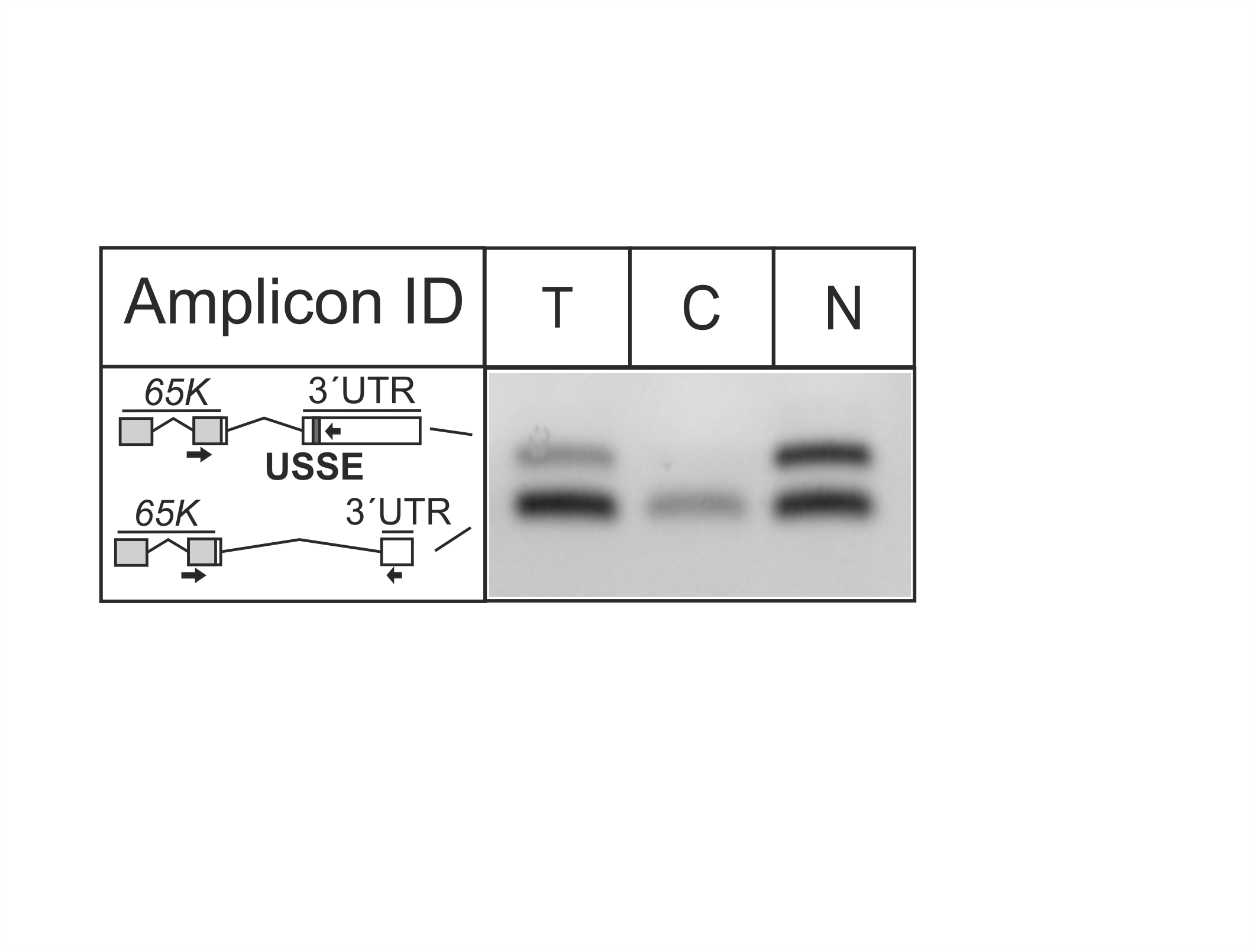

Supplement: S6 Fig — Following cellular fractionation of HEK293 cells, multiplex RT-PCR was performed to measure levels of 65K long and short isoforms (T: total fraction, C: cytoplasmic fraction, N: nuclear fraction). Black arrows show primer location: forward primer targets both isoforms, while reverse primers target either long or short isoform. (TIF) [file pgen.1006824.s006.tif]

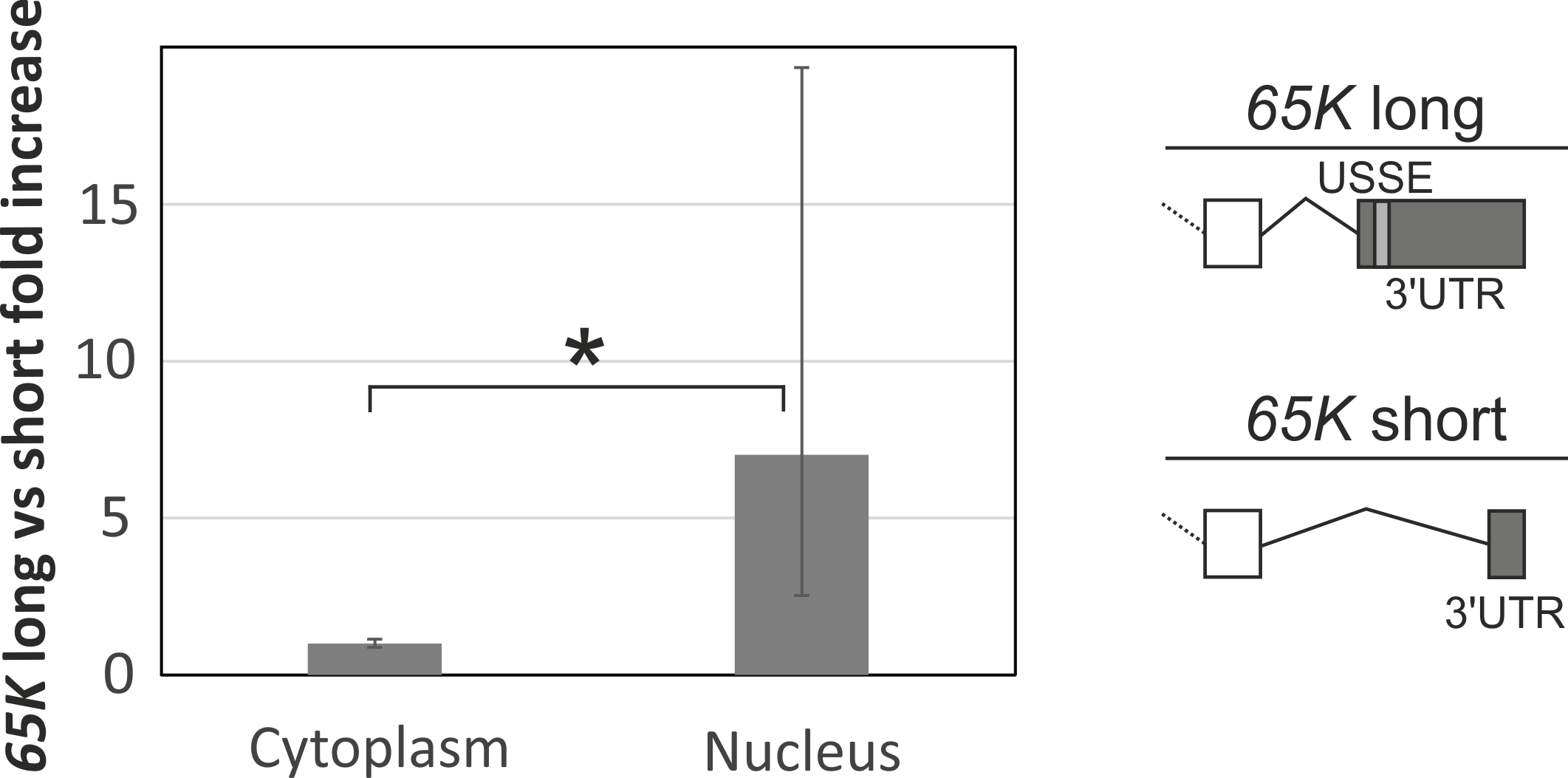

Supplement: S7 Fig — Following cellular fractionation of CHO cells, qRT-PCR was performed to measure levels of 65K long and short isoforms. 65K long versus short isoform ratios were compared in cytoplasmic and nuclear fractions. Error bars represent standard deviation for 3 biological replicates and the asterisk indicates p-value < 0.05 in two-tailed Student′s t-test. (TIF) [file pgen.1006824.s007.tif]

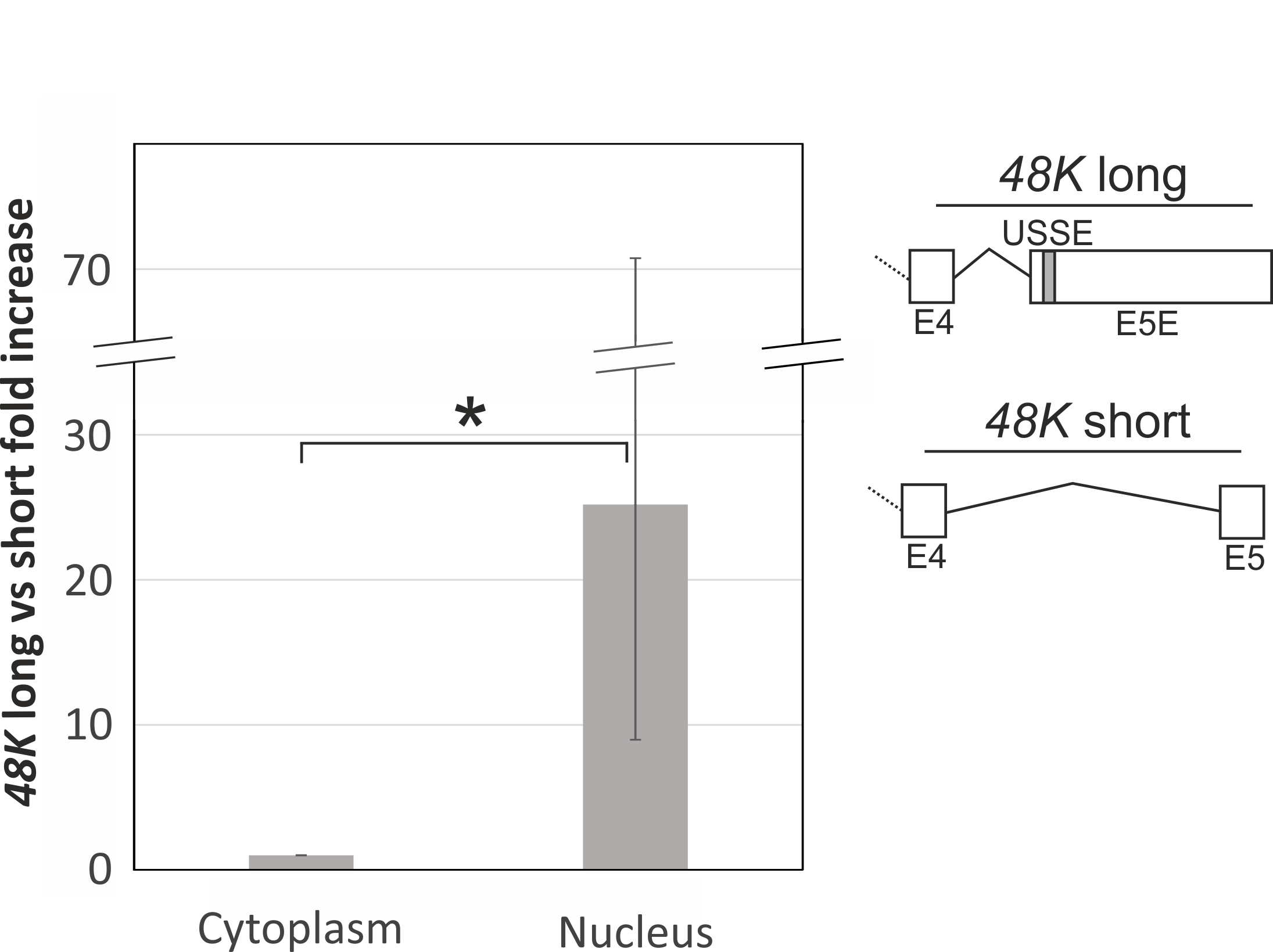

Supplement: S8 Fig — Following cellular fractionation in CHO cells, qRT-PCR was performed to measure levels of 48K long and short isoforms. The 48K long isoform contains E5E, an extended exon that results from USSE-directed intron retention. 48K long versus short isoform ratios were compared in cytoplasmic and nuclear fractions. Error bars represent standard deviation for 3 biological replicates and the asterisk indicates p-value < 0.05 in two-tailed Student′s t-test. (TIF) [file pgen.1006824.s008.tif]

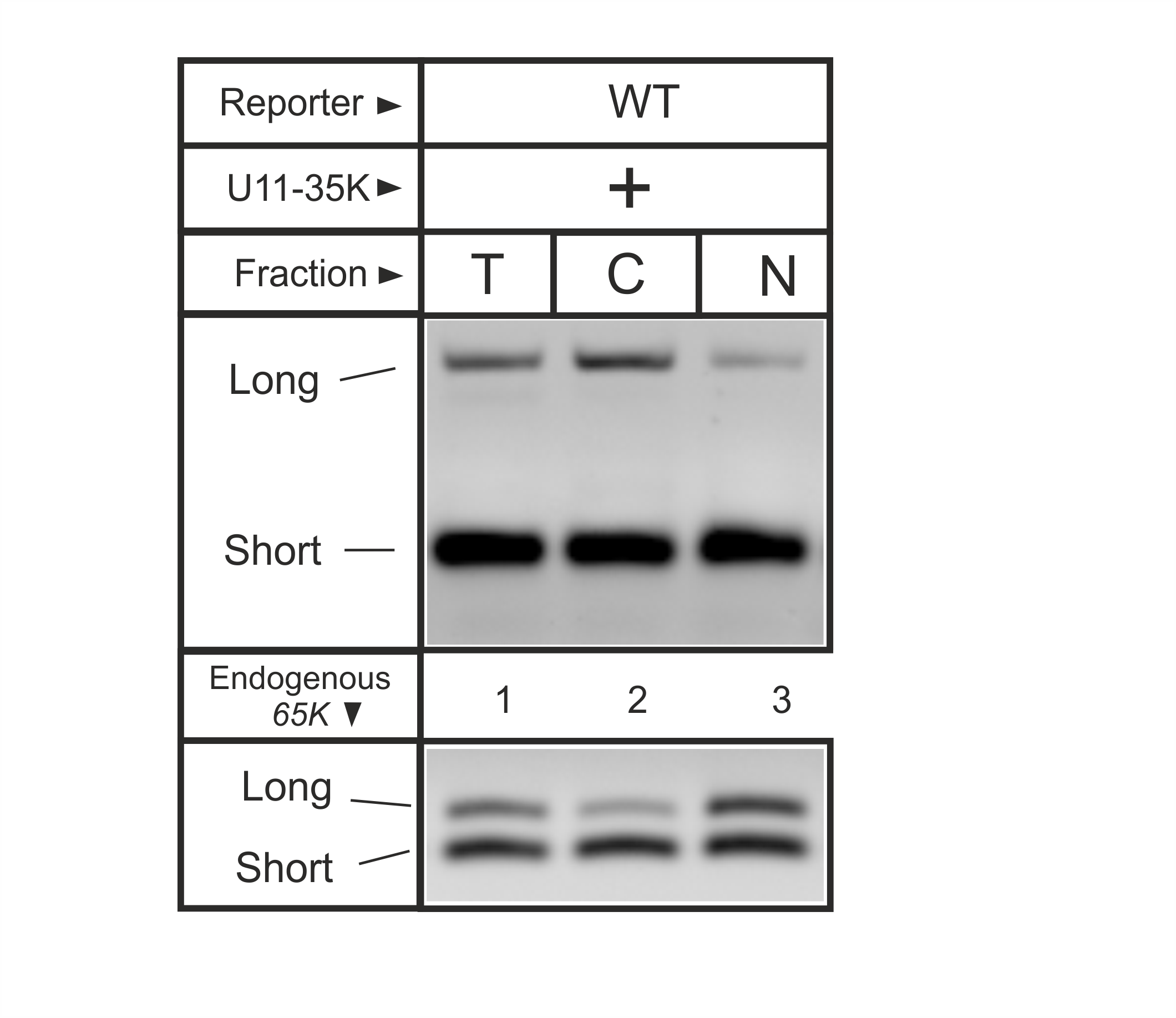

Supplement: S9 Fig — HeLa cells were transfected with HP reporters and λN peptide-fused U11-35K. After cellular fractionation, distribution of construct-derived long and short isoform was assayed through RT-PCR. T is total, C is cytoplasmic and N is nuclear fraction. Endogenous 65K isoform cellular distribution served as fractionation quality control. (TIF) [file pgen.1006824.s009.tif]

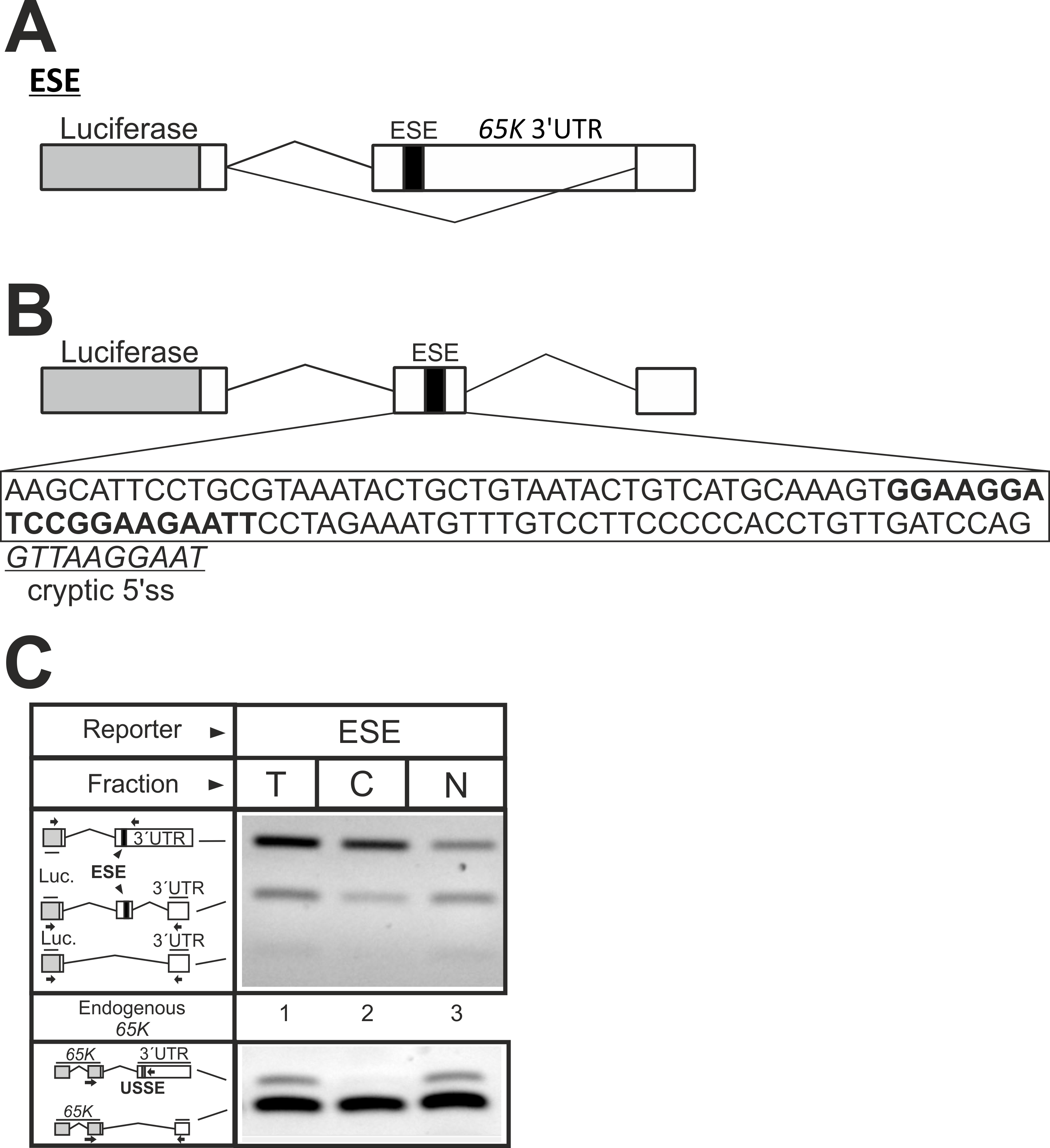

Supplement: S10 Fig — (A) Schematics of the ESE construct, in which the USSE was replaced with an ESE [78]. (B) Sequence of the cryptic splicing isoform with the ESE sequence in bold. (C) Multiplex RT-PCR analysis of HeLa cells transfected with the ESE. The identities of the long, short and the cryptic splicing isoforms were confirmed by sequencing. (TIF) [file pgen.1006824.s010.tif]

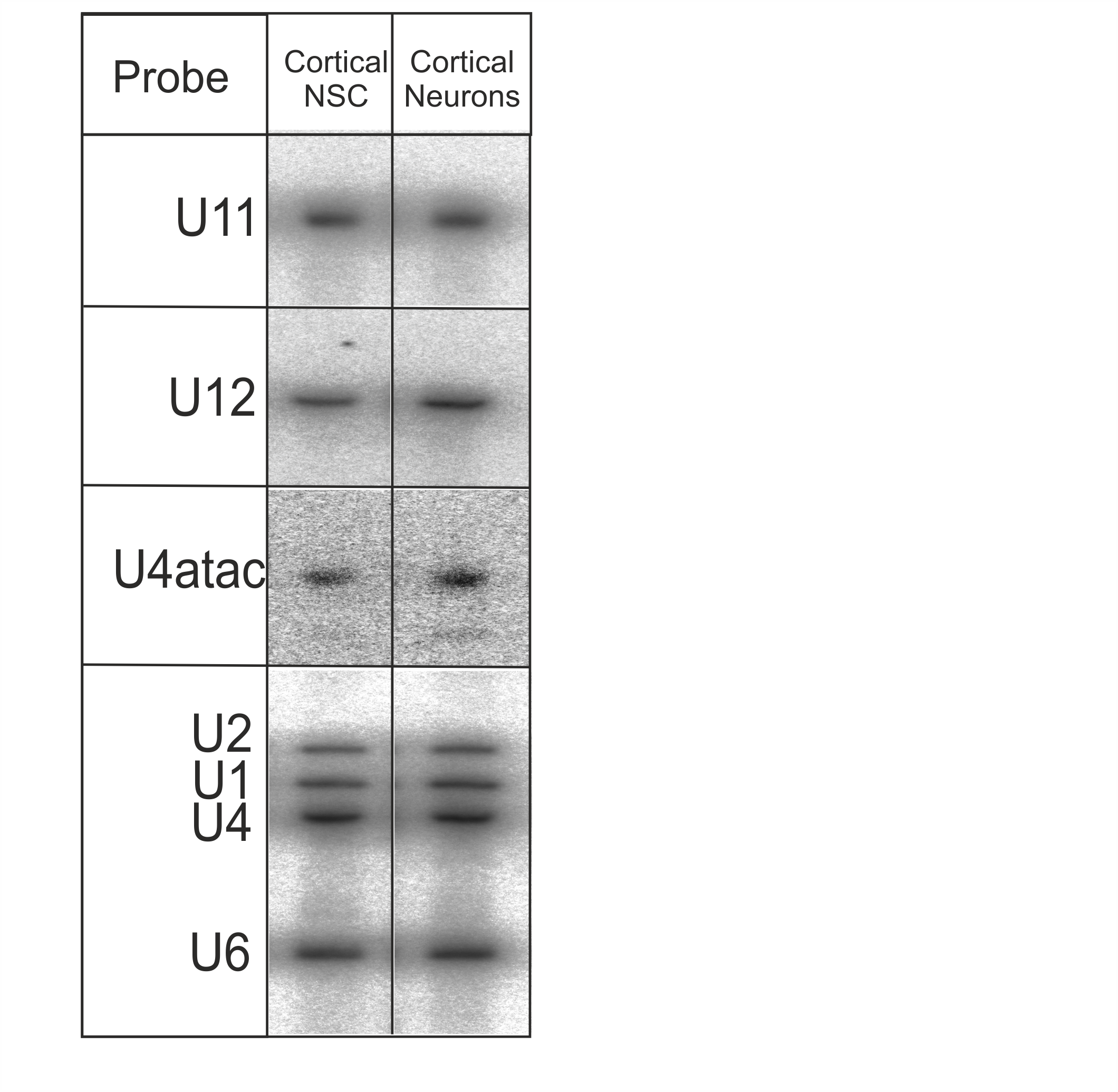

Supplement: S11 Fig — Northern blot analysis of indicated mouse cells. One μg of RNA was separated on an 8% polyacrylamide gel, blotted on a nylon filter and sequentially probed for U11, U12 and U4atac snRNAs and major spliceosomal snRNAs, respectively. (TIF) [file pgen.1006824.s011.tif]

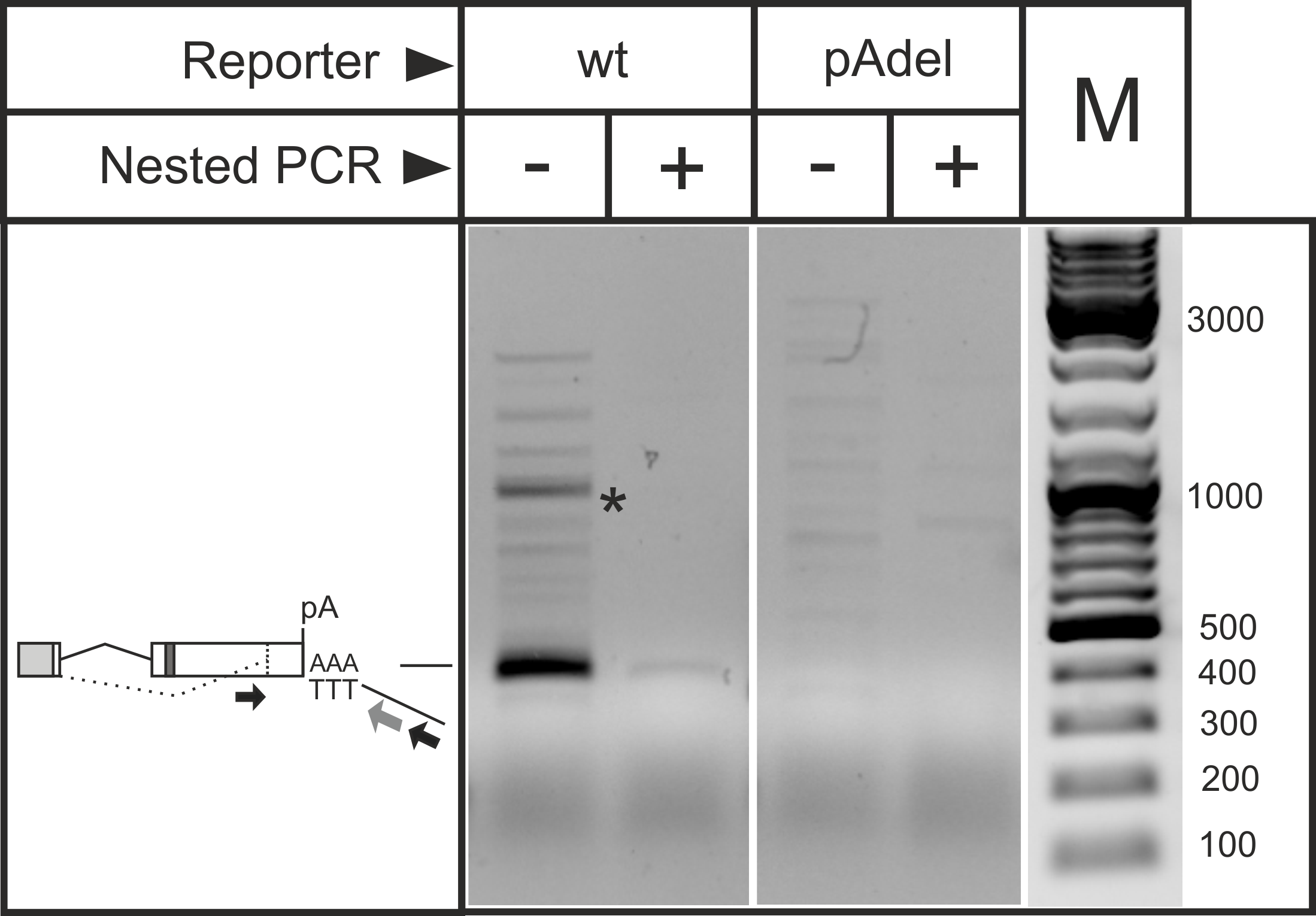

Supplement: S12 Fig — Wt and pAdel constructs were transfected in CHO cells, and 3′RACE was performed to analyze alternative polyadenylation. The pAdel construct served as a negative control. The asterisk indicates a non-specific amplicon as both nested PCR and the pAdel construct did not reveal a similar amplicon. Arrows indicate primer location and the grey arrow the position of the nested reverse primer. The marker (M) is Generuler (Thermo Scientific: #SM0331). (TIF) [file pgen.1006824.s012.tif]
