# Supplementary material for: Alternative exon definition events control the choice between nuclear retention and cytoplasmic export of U11/U12-65K mRNA
Source: PLoS Genet. 2017 May 26;13(5):e1006824. doi: 10.1371/journal.pgen.1006824 (PMC5473595; doi:10.1371/journal.pgen.1006824)
Supplement: S1 Table — (DOCX) [file pgen.1006824.s013.docx]

| S1 Table. EST, mRNA, or RNAseq sequences supporting *RNPC3-Amy2A/1* conjoined gene. | | |
| --- | --- | --- |
| Species | **Accession number** | **Database** |
| *Bos taurus* | EH140677, EE246812 | www.ncbi.nlm.nih.gov/genbank/ |
| *Canis familiaris* | DN440643 | www.ncbi.nlm.nih.gov/genbank/ |
| *Homo sapiens* | AK095605.1, BC011179 | www.ncbi.nlm.nih.gov/genbank/ |
| *Macaca fascicularis* | AB173575 | www.ncbi.nlm.nih.gov/genbank/ |
| *Mus musculus* | CO425071.1 | www.ncbi.nlm.nih.gov/genbank/ |
| *Rattus norvegicus* | HWI-ST753:124:C0WVNACXX:3:1307:4112:181124  HWI-ST753:124:C0WVNACXX:5:1301:5504:70429 | http://rgd.mcw.edu/jbrowse/?data=data_rgd6&loc=Chr2%3A216481129..216482568&tracks=ARGD_curated_genes%2CPhenogen_BNLx_Brain%2CPhenogen_SHR_Brain&highlight= |
