# Supplementary material for: Alternative exon definition events control the choice between nuclear retention and cytoplasmic export of U11/U12-65K mRNA
Source: PLoS Genet. 2017 May 26;13(5):e1006824. doi: 10.1371/journal.pgen.1006824 (PMC5473595; doi:10.1371/journal.pgen.1006824)
Supplement: S2 Table — (DOCX) [file pgen.1006824.s014.docx]

| **S2 Table.** **Minor spliceosome protein-encoding transcripts monotonically down- or up-regulated during neuronal differentiation^a^**^)^ | | | | | | | |
| --- | --- | --- | --- | --- | --- | --- | --- |
| **Gene name** | **Transcript ID** | **Transcript type** | **Avearage expression, TPM^b)^** | **Relative amplitude of change^c)^** | **Kendall rank correlation coefficient, τ** | **Kendall, P-value** | **Kendall, BH-adjusted P-value** |
| Rnpc3 | ENSMUST00000106535.1 | protein_coding | 2.32 | 0.96 | 0.43 | 1.11E-03 | 4.22E-03 |
| Rnpc3 | ENSMUST00000106536.7 | protein_coding | 10.44 | 0.72 | -0.52 | 9.10E-05 | 6.92E-04 |
| Snrnp48 | ENSMUST00000178564.1 | protein_coding | 4.78 | 0.76 | -0.44 | 8.69E-04 | 4.13E-03 |
| Zcrb1 | ENSMUST00000161409.1 | nonsense_mediated_decay | 8.51 | 0.79 | 0.61 | 4.17E-06 | 5.35E-05 |
| Zrsr2 | ENSMUST00000112289.8 | protein_coding | 4.15 | 0.80 | -0.68 | 2.62E-07 | 9.94E-06 |
|  |  |  |  |  |  |  |  |
| a) The table shows significantly down- or up-regulated transcripts (BH-adjusted p<0.005; relative amplitude of change ≥0.7) expressed at readily detectable levels (average TPM value ≥2) during differentiation of mouse ES cells into glutamatergic neurons in vitro. | | | | | | | |
| b) TPM is the transcript per million expression value calculated by Kallisto. | | | | | | | |
| c) Relative amplitude of change is calculated as a difference between maximum and minimum expression values normalized to the maximum. | | | | | | | |
