# Supplementary material for: Alternative exon definition events control the choice between nuclear retention and cytoplasmic export of U11/U12-65K mRNA
Source: PLoS Genet. 2017 May 26;13(5):e1006824. doi: 10.1371/journal.pgen.1006824 (PMC5473595; doi:10.1371/journal.pgen.1006824)
Supplement: S3 Table — (DOCX) [file pgen.1006824.s015.docx]

| **S3 Table: Oligonucleotides used in this study** | | | | |
| --- | --- | --- | --- | --- |
| **Name** | **Purpose** | **Target** | **See Figures** | **Sequence (5′-3′)^1^** |
| h65K-11 | RT-PCR | human *65K* gene | 2A, S4 | CCATGGTGGTTCAGTTTGCT |
| h65K-79 | RT-PCR | human *65K* long isoform | 2E, 3E, 4G, 5A, 5C, 5D, S4, S6, S9, S10 | GGGGGAAGGACAAACATTTC |
| h65K-88 | RT-PCR, qPCR | human *65K* gene | 2E, 3C, 3E, 4G, 5A, 5C, 5D, S6, S9, S10 | CCAGGTGGTCAATTTCTTCC |
| h65K-121 | RT-PCR, qPCR | luciferase gene | 2D, 2E, 2G, 3E, 3G, 4I, 5A, 5C, 5D, S5, S9, S10 | CAAGAAGGGCGGCAAGAT |
| h65K-123 | RT-PCR, qPCR | human *65K* long isoform | 2D, 4I | TTTGCATGACAGTATTACAGCAG |
| h65K-145 | RT-PCR, qPCR | human *65K* gene | 2D, 4I, S4 | CCAGGTGGTCAATTTCTTCC |
| h65K-168 | oligo for cDNA (α) | human *65K* gene | S4 | AGTTCCATAGTCC |
| h65K-171 | oligo for cDNA (β) | human *65K* gene | S4 | TCGCCAGATTCAA |
| h65K-172 | oligo for cDNA (γ) | human *65K* gene | S4 | TCAGCCCTCCATA |
| h65K-173 | RT-PCR | human *65K* gene | S4 | CACTTGAAGGACACGGGATT |
| h65K-175 | RT-PCR | human *65K* gene | 2A | AGTTCCATAGTCCAGGTGGTCA |
| h65K-177 | RT-PCR | *65K-AMY2B* intergenic exon | 2A | CCACATCGCCAGATTCAAC |
| h65K-193 | 3’RACE | Oligo dT adapter for 3’RACE | S12 | GCGAGCACAGAATTAATACGACTCACTATAGGTTTTTTTTTTTTTTTT |
| h65K-194 | 3’RACE | anchor primer 1 for 3’RACE | S12 | GCGAGCACAGAATTAATACGACT |
| h65K-195 | 3’RACE | anchor primer 2 for 3’RACE | S12 | GAATTAATACGACTCACTATAGG |
| h65K-196 | RT-PCR | human *65K* gene | 4G, 5A, 5C, 5D, S6, S9, S10 | GCTCGATCTGCTAGACCAAAA |
| h65K-199 | 3’RACE | human *65K* long isoform | S12 | TCAGTTTCTTTATAAGCGGTTTATTT |
| h65kK-200 | RT-PCR, qPCR, 3’RACE | human *65K* long isoform | 2E, 2F, 2G, 3C, 3E, 3F, 3G, S12 | AAATGGTTCAGCTCTTAGTTATTTTT |
| h65K-211 | qPCR | *65K-AMY2B* intergenic exon | 2B | GTTGAATCTGGCGATGTGG |
| h65K-212 | RT-PCR, qPCR | *65K-AMY2B* intergenic exon | 2B, 2E, 2F, 3C, 3E, 3F, S5 | CTGGGATGAAAGATCACTTGAA |
| h65K-219 | cloning | pGL4.13-F65KpA |  | CACAACAGAAAGCATATGTTATTGAA |
| h65K-220 | cloning | pGL4.13-F65KpA |  | CCATGGACGCGTGCAGACATGATAAGATACATTGATGAG |
| h65K-221 | cloning | *65K*, genomic DNA |  | AATTCAATAACATATGCTTTCTGTTG |
| h65K-224 | cloning | *65K*, genomic DNA |  | CCATGGACGCGTTTCCCAGTATCCATACCTATATAAAGTC |
| h65K-230 | cloning | *65K*, genomic DNA |  | TCTTCGGCATCAAAAACACATTGTGCATGTTGGGTACAGG |
| h65K-232 | cloning | *65K*, genomic DNA |  | CCTGTACCCAACATGCACAATGTGTTTTTGATGCCGAAGA |
| h65K-246 | T7 transcription template | pGL4.13-F65KpA |  | GCGAAGCTTAATACGACTCACTATAGGGAGAGATGTGTTAAACCAGATAAAGAAAAA |
| h65K-257 | T7 transcription template | pGL4.13-F65KpA |  | AAATGGTTCAGCTCTTAGTTATTTTT |
| h65K-313 | RT-PCR | construct short-3’UTR isoform | 2E, 3E | GCCGTGTAATAATTCTAGAATTAATAAAGGT |
| h65k-353 | RT-PCR | downstream region of human *65K* gene | 2E, 2G, 3E, 3G | GATGTGTTAAACCAGATAAAGAAAAA |
| h65K-376 | cloning | ESE construct-*65K* long isoform | S10 | TTCCTAGAAATGTTTGTCCTTCC |
| h65K-377 | cloning | ESE construct-*65K* long isoform | S10 | TTCTTCCGGATCCTTCCACTTTGCATGACAGTATTACAGCAG |
| h65K-428 | RT-PCR | human *65K* long isoform | 5A, 5C, 5D, S9, S10 | TGAACACATTCTCTAAGTAAGTTGACC |
| h65K-456 | RT-PCR | construct short-3’UTR isoform | 2F, 3F |  |
| hTPS1-1 | RT-PCR | human TPS1 gene | 4I | CGCCATCAGGGTAAAGAACT |
| hTPS1-2 | RT-PCR | human TPS1 gene | 4I | GGGGTTGGTGTCCCAGTAG |
| h65K-Mor1 | USSE block morpholino | *65K* pre-mRNA | 2A, 2B, 3B, 4D, 4E, 4F | AAAGGATACGACAAGAAAGGATACA |
| h65K-Mor2 | Mock morpholino | *65K* pre-mRNA | 2A, 2B, 3B, 3C, 4B, 4C, 4F | TTCTCCCATCTCGGACTTGCAAAGT |
| h65K-Mor3 | U1 block morpholino | *65K* pre-mRNA | 3B, 3C | CAAAGCTGTTACGCACAGTTCCATA |
| m65K-1 | RT-PCR | mouse *65K* pre-mRNA | 1D, 1F | TTGACCCTCTGGACTTTGGA |
| m65k-4 | RT-PCR | mouse *65K* pre-mRNA | 1D, 1F | CCACTCAAACAGGTGGACAA |
| m65k-5 | RT-PCR | mouse *65K* pre-mRNA | 1D , 1E | TCTGCTAGACCAAAACAGGATT |
| m65k-6 | RT-PCR | mouse *65K* pre-mRNA | 1D, 1E | CAACGCACTTCTAGGAAACAAA |
| m65k-7 | RT-PCR | mouse *65K* pre-mRNA | 1D, 1E | TCCAAAGTCCAGAGGGTCAA |
| mGAPDH-3 | RT-PCR | mouse *GAPDH* | 1D | AGGTCGGTGTGAACGGATTTG |
| mGAPDH-4 | RT-PCR | mouse *GAPDH* | 1D | TCAATGAAGGGGTCGTTGAT |
| Renil-3 | RT-PCR, qPCR | pGL4.73 Renilla | 2E, 3E | CCTCTAGGTCTTGGGGGAAG |
| Renil-4 | RT-PCR, qPCR | pGL4.73 Renilla | 2E, 3E | TCCAGGCCTTGCTTCTCTAA |
| ^1^DNA uppercase, Morpholino uppercase underlined | | | | |

*h65K*
