# Supplementary material for: Alternative exon definition events control the choice between nuclear retention and cytoplasmic export of U11/U12-65K mRNA
Source: PLoS Genet. 2017 May 26;13(5):e1006824. doi: 10.1371/journal.pgen.1006824 (PMC5473595; doi:10.1371/journal.pgen.1006824)
Supplement: S4 Table — (DOCX) [file pgen.1006824.s016.docx]

| **S4 Table: smFISH probes used in this study** | |
| --- | --- |
| **Probe set** | **Sequence (5′-3′)** |
| **TOTAL set** | ATCCCCTTGATATCGCAAGC CTGACCAGAAGGGTTCGGTC CTTTCTCCTCAGCAGTAAGC CCGAAGTACTTCAGCAAGTC CTTATCTGACAGGACCCGCA AAGCTGTATGTTTCAGTCGC AGCTGCTTTTTCATTAGGGA GGAGTCTTGTCAATGCCTTT TGACCTAAAAGTTTCAGTTG TGCAAATTCAACGACTAAAG GAGTGAACTCGATCTTGCTC TTTTTCAGAGCCTGAAGTGG TATCATCTTCGACAGGGTCA TTGGTGCAATTCCATTTTCT TTAAAGGAAAAGTCAGCCCA GGGTACATATACTTGAGGCA CATTTACAATGTTTGCTAGG AACTTAGGCACGCTTGCCAA AAGATGAAGGACCTGTACAT TGGGCAAATTCATTTTATTC CGCAGTAATTGGTCCAAAAG TGCATGCAATGGCATATAGT AATTCCTCATCCTCGTCTGG TCAGTGCTTTCATATTCTGA TTGTTCATTCTCTGTCGGTC CTTTTGGGCTGAAGATTTGC GGCGCTGCTTTATTGTTTTA AGGACACAAAGGTGTATTCA AGCACTGGATGTAAACTGTG GGTCAAATACATCTGAAGGT TTGTTACCTACAGGTTGTGG TTCTTAAATGCAGCTGGCAT TGCATCAACTTCAGTAGCAG GGGAAGATTTTTCCAAATCC ATCTCCTCTGTGATGTCCAA TGAAGGCATTTCATCAGAGT ACTGAAAGTGTTTCCATTTC CACCCGGTTCATAACTTCTG ACATAAATTCTACAGTTTGG GGTCCTTTTCTTGAACATGT TTCTGATGAAAAGTCAACAT TATCAAACATGATCCGCTGT TCCAATGAAAGCTTGTCCTT GCTGCTGCTTTTTCATTAGG CCATTAGCTTCCTTTAAGGC CCATGGGTTTTCCAAAAAGC |
| **LONG set** | CAGCAGTATTTACGCAGGAA AGGATACACTTTGCATGACA CCCCAAAAAGGATACGACAA AAGGACAAACATTTCTAGGA CCTTAACCTGGATCAACAGG AGATCTGTTCAGTCTATACA AGGACAGCACAGAAAATTCA AGTTGACCTGATTCACTAAC ACAAGACTGTATTCTGGGTT ACACAACAGAAAGCATATGT GAATTTACATAGTAATCCAG ATGAGAGGTCTGCTACTCTC TGTCAAAAGAGATTCACTAA AGAAAGATACTTCTATTGCT GCTTTTCTTCTCTTGTTTAG ACTACCCTTAGTGGGCATTA TGCCAAGACTGTTTTTCCTT GTCTGGAAACCACTATTCCT TGGTGATTACGGTCATCAAC GCAGTGTCTAAACTGGTCAG AGGTTGATACTAGGTACCTA GAAACACAGAGGCTCCAACC TAGTAGCAGATTTTTCAACA GTGATAATAACCGAATTTTC ACCGCTTATAAAGAAACTGA TGAATCATGTCAGTATAACA AACTAAGAGCTGAACCATTT ACACAGGTCATTAGTTTAAA GCCCATTAAAAATAGGAAGT |
